# A genome‐wide association study identifies novel association between genetic variants in *GGT7* and *LINC00944* and hypertension

**DOI:** 10.1002/ctm2.388

**Published:** 2021-05-21

**Authors:** Chengcheng Tan, Hongfu Zhang, Dong Yu, Yao Hu, Pengxia Wang, Dan Wang, Jingjing Fa, Han Ran, Xiaoyu Zhang, Yanming Chen, Weixi Qin, Chen Fang, Tie Ke, Nianguo Dong, Jianping Cai, Qing He, Shaofeng Huo, Junhan Wang, Xiang Ren, Xin Tu, Xu Lin, Qing Wang, Chengqi Xu

**Affiliations:** ^1^ College of Life Science and Technology and Center for Human Genome Research Huazhong University of Science and Technology Wuhan P.R. China; ^2^ University‐Affiliated Hospital Huazhong University of Science and Technology Wuhan P.R. China; ^3^ Department of Cardiovascular Surgery Union Hospital Tongji Medical College Huazhong University of Science and Technology Wuhan P.R. China; ^4^ Key Laboratory of Systems Health Science of Zhejiang Province Hangzhou Institute for Advanced Study University of Chinese Academy of Sciences, Chinese Academy of Sciences Hangzhou China; ^5^ Shanghai Institute of Nutrition and Health University of Chinese Academy of Sciences, Chinese Academy of Sciences Shanghai China; ^6^ Key Laboratory for Biorheological Science and Technology of Ministry of Education Bioengineering College of Chongqing University Chongqing P.R. China; ^7^ Key Laboratory of Geriatrics, Ministry of Health Beijing Hospital Beijing P.R. China; ^8^ Department of Cardiology Beijing Hospital Beijing P.R. China

Dear Editor,

Hypertension affects one billion people in the world.^1^ Half of the Chinese population aged from 35 to 75 years is also affected with hypertension.^2^ Genetic factors contribute to hypertension. Different ethnic populations share some common genetic factors; however, population‐specific genetic factors also play important roles in common complex diseases in different ethnic populations. Most genome wide association study (GWAS) for blood pressure have been reported in European ancestry populations; thus, much more GWAS are needed for hypertension in non‐European ancestry populations, including the Chinese population.

We designed a three‐phase GWAS as reported previously^3^ to identify novel genomic variants conferring risk to hypertension in the Chinese Han population. The overall study design is shown in Figure 1A. Genotyping, imputation, and principal component analyses were conducted in phase 1 GWAS samples with 353 cases with hypertension and 332 controls without hypertension (Figure S1). After quality control, 3,956,088 single nucleotide polymorphisms (SNPs) were analyzed for their association with hypertension with adjustment of age, age^2^, gender, and the first three principal components (Figure S2). A total of 17,435 SNPs showing *p* value of <5.0 × 10^–3^ were selected for the phase 2 in silico replication study using GWAS summary data from the NHAPC cohort (1592 cases and 1302 controls).^4^ One hundred thirty‐six SNPs clustering into 15 independent loci (Table S2) showed nominal association with hypertension (*p *< 5 × 10^–3^). Sixteen leading SNPs representing 15 loci were selected for replication in the phase 3 population containing 3274 cases and 2734 controls. Fourteen SNPs at 13 loci were genotyped successfully (two of 16 SNPs failed in genotyping). Two SNPs, including rs10847208 in the last exon of a long noncoding RNA (lncRNA) gene *LINC00944* on 12q24.32 and rs2064453 in the promoter of *GGT7* encoding gamma‐glutamyltransferase 7 on 20q11.22, showed significant association with hypertension with *P*_adj_ of 3.31 × 10^–3^ (odds ratio [OR] = 1.20) and 2.98 × 10^–3^ (OR = 1.14), respectively, after Bonferroni correction for multiple testing (Figure 1B, Table S3). Analysis of the GWAS summary statistics for hypertension in the UK Biobank^5^ (https://pan.ukbb.broadinstitute.org.) showed that rs2064453 was associated with essential hypertension in the East Asian population (*p *= 0.004) and the European population (*p *= 0.01). SNP rs2064453 also showed positive association with diastolic blood pressure in 417,003 European individuals (*p* = 0.0055 with combined_medadj_raw).

**FIGURE 1:**
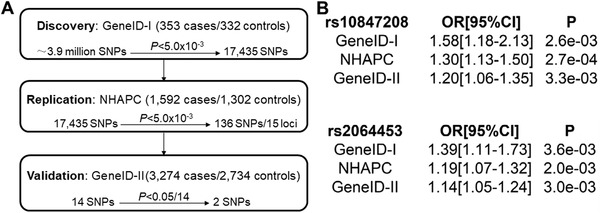
Overall GWAS design and identification of two new loci for hypertension in a Chinese population. (A) Overall design of a three‐stage GWAS for hypertension in a Chinese population (discovery‐GeneID‐I, replication‐NHAPC, and validation‐GeneID‐II). (B) Association signals are shown for each of three Chinese populations GeneID‐I, NHAPC, and GeneID‐II. Abbreviations: CI, confident interval; OR, odds ratio

SNP rs2064453 is located within a CpG island in the promoter of *GGT7* and in a DNase I Hypersensitivity Peak Cluster (Figure 2A). From the GTEx data, the TT and TC genotypes of rs2064453 showed significantly higher expression of *GGT7* than the CC genotype in 12 tissues. We also performed eQTL (expression quantitative trait locus) analysis in blood leucocytes from 309 Chinese study subjects, and significant eQTL was identified between rs2064453 and *GGT7* under a dominant model (TT+TC > CC, *p *= 0.003), an additive model (TT > TC/CC, *p *= 0.001), and a recessive model (TT > TC+CC, *p *= 0.018) after adjusting for age and gender (Figures 2B–2D). The data suggest that risk allele T of rs2064453 increases risk of hypertension by increasing the expression level of *GGT7*. SNP rs2064453 did not show association with the expression levels of nearby genes, including *GSS*, *ACSS2*, *MAP1LC3A*, and *NCOA6* (Figure S3). To determine how risk allele T of rs2064453 increases the expression level of *GGT7*, we cloned a 2 kb *GGT7* promoter and regulatory region containing either the C allele or T allele of rs2064453 into the pGL3‐basic luciferase reporter vector (Figure 2E). Luciferase assays showed that the T allele had a significantly higher luciferase activity than the C allele (Figure 2F). Together with the eQTL data, these data suggest that risk allele T of rs2064453 can increase expression of *GGT7* by enhancing the transcription activation of *GGT7*.

**FIGURE 2:**
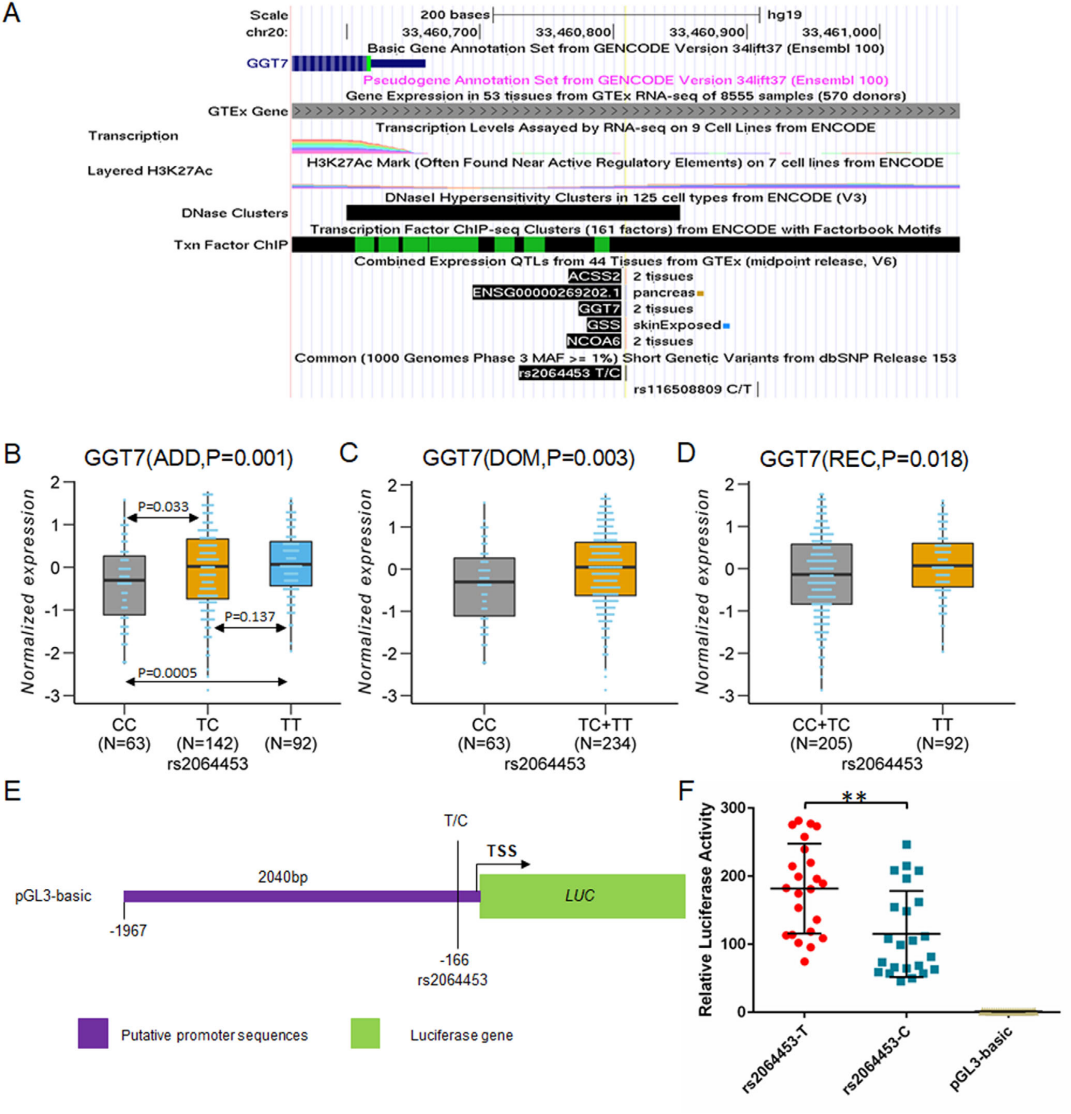
Risk allele T of the lead variant rs2064453 at the new hypertension locus on chromosome 20q11.22 shows association with upregulation of *GGT7* and significantly increases transcription activation from *GGT7* promoter. (A) SNP rs2064453 is located within a CpG island in the promoter/regulatory region of *GGT7*, and a DNase I Hypersensitivity Peak Cluster (http://genome.ucsc.edu/cgi‐bin/hgTrackUi?db = hg19&g = wgEncodeRegTfbsClusteredV3). (B‐D) Significant eQTL of variant rs2064453 with *GGT7* under an additive, dominant, or recessive model. The number of study subjects is indicated with N. (E) Luciferase reports pGL3‐Basic‐rs2064453‐T and pGL3‐Basic‐rs2064453‐C with the 2040 bp *GGT7* promoter/regulatory region with wither risk allele T and allele C cloned upstream of the firefly luciferase gene in the pGL3‐basic vector. SNP rs2064453 is located at ‐166 bp from the transcriptional start site (TSS) of *GGT7* gene. (F) Luciferase assays showing that risk allele T of rs2064453 promotes a significantly more transcription activation than allele C (*n* = 23). Empty pGL3‐basic vector was used as a negative control. **p < 0.01. Line is for mean with SD. *p* value of B‐D was obtained by linear regression after adjustment with age and gender. *p* value of F was obtained by Student's *t* test

*GGT7* encodes an extracellular gamma‐glutamyl transferase and acts as an extracellular enzyme. Thus, we purified 6xHis‐tagged GGT7 protein and used it to treat endothelial cells (EA.hy926). Compared with control, 30 min treatment with the GGT7 protein significantly reduced the level of phosphorylation of ERK1/2 in endothelial cells (Figures 3A and 3B). Moreover, GGT7 treatment consistently reduced ERK1/2 activation at different time points of 0.5, 2, 7, 22, 27, and 31 h (Figures 3C and 3D). A recent finding showed that both the systolic and diastolic blood pressure was increased in mice deficient of *Erk1* and *Erk2* in endothelial cells.^6^ These data suggest that *GGT7* variant rs2064453 increases risk of hypertension by reducing ERK1/2 activation.

**FIGURE 3:**
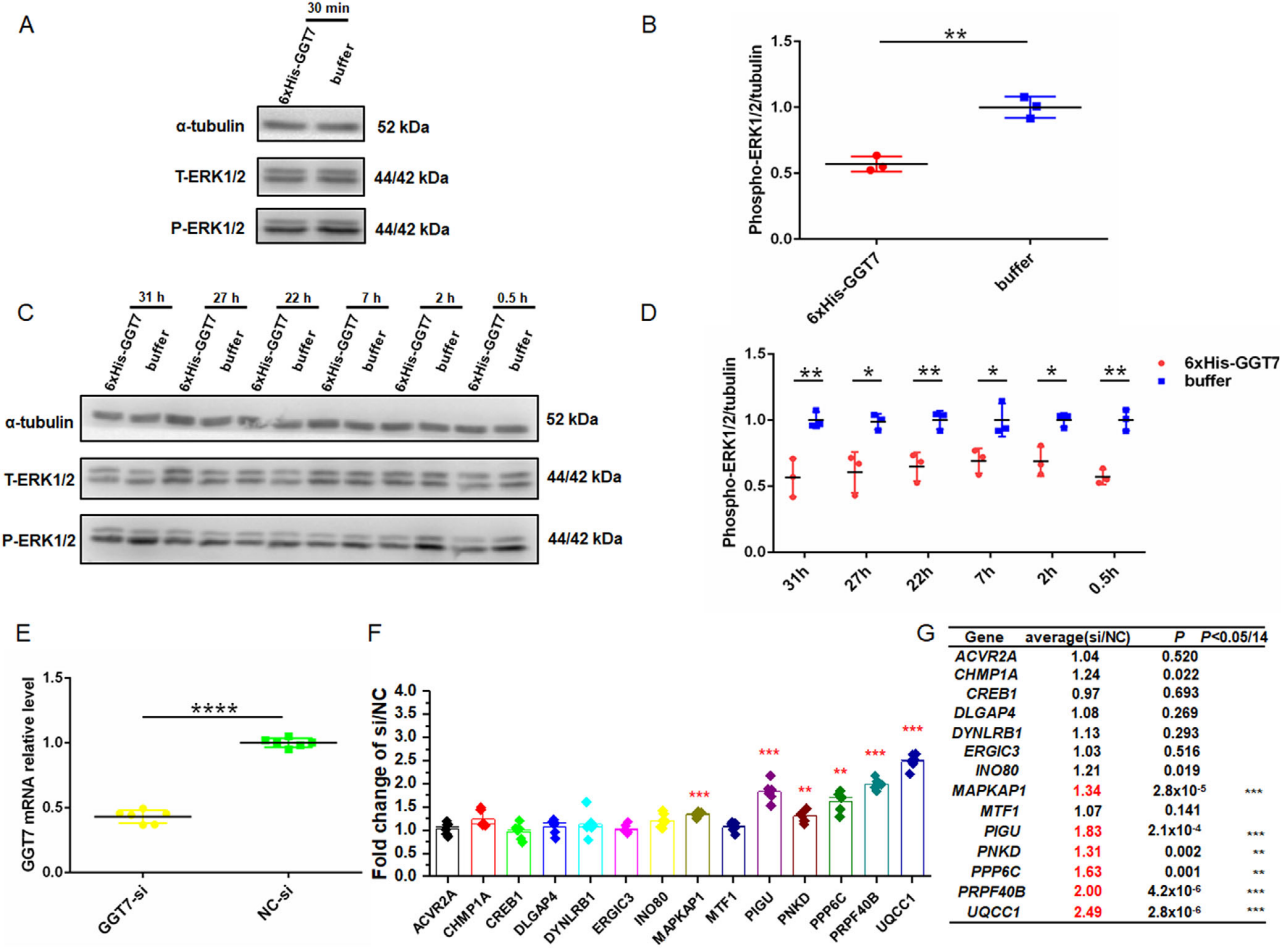
GGT7 protein activates ERK1/2 signaling and identification of downstream genes regulated by *GGT7*. (A) Western blot analysis showing that treatment of EA.hy926 endothelial cells with the GGT7 protein for 30 min decreases ERK1/2 phosphorylation. (B) Western blot images as in (A) were scanned, quantified and plotted (*n* = 3). (C) Western blot analysis showing that GGT7 consistently reduces ERK1/2 activation at different time points of 0.5, 2, 7, 22, 27, and 31 h. T‐ERK1/2, total ERK1/2; P‐ERK1/2, phosphorylated ERK1/2 at Thr202/Tyr204. (D) Western blot images as in (C) were scanned, quantified, and plotted (*n* = 3). (E) Real‐time reverse transcription‐polymerase chain reaction (RT‐PCR) analysis showing successful knockdown of *GGT7* by transient transfection of EA.hy926 ECs with *GGT7* siRNA (GGT7‐si) compared with negative control siRNA (NC‐si) (*n* = 6). (F) Real‐time RT‐PCR analysis showing that knockdown of *GGT7* expression significantly increased the expression of *MAPKAP1*, *PIGU*, *PNKD*, *PPP6C*, *PRPF40B*, and *UQCC1*. (G) Summary data from real‐time RT‐PCR analysis as in (F). **p < 0.01, ***p < 0.001. Line is for mean with SD. *p* value was obtained by Student's *t* test

We also used HumanBase database^7^ to identify candidate genes whose expression or function is linked to GGT7 and validated them in EA.hy926 endothelial cells. Knockdown of *GGT7* with siRNA affected the expression of *MAPKAP1*, *PIGU*, *PNKD*, *PPP6C*, *PRPF40B*, and *UQCC1* (Figure 3). Importantly, knockdown of *GGT7* significantly increased the expression level of *PPP6C* by 1.63 fold (*p *< 0.01) (Figure 3C). Recently, Li et al showed that conditional knockout mice for *Ppp6c* in T cells showed significantly increased systolic and diastolic blood pressure upon induction with angiotensin II.^8^ These data suggest that *GGT7* variant rs2064453 increases risk of hypertension by reducing *PPP6C* expression, too.

SNP rs10847208 shows a sQTL (splice quantitative trait locus) with *LINC00944* (normalized effect size = 0.41, *p *= 7.8 × 10^–7^) in testes in the GTEx Portal database (Figure S3B). Meanwhile, we explored the effect of rs10847208 on a potential enhancer activity or binding of potential microRNA by cloning a 201 bp genomic DNA fragment with either allele C or T in the middle into pGL3‐promoter and pMIR luciferase reporter, respectively; however, no effect was observed for rs10847208 (Figures S3 and C‐F). Further studies are needed to determine how SNP rs10847208 increases risk of hypertension.

The NHAPC cohort, that is, the phase 2 replication population for our GWAS, was also used as one of the six cohorts in a previous GWAS for blood pressure in the Chinese population by Lu et al.^9^ Comparison between the two GWAS identified one potential overlapping locus for hypertension at *FSTL5*. We found that SNP rs28587458 in intron 11 of *FSTL5* showed positive association with hypertension in the discovery population (*p *= 3.7 × 10^–3^), the replication population (*p *= 4.1 × 10^–3^), and the combined population (*p_meta_* = 5.9 × 10^–4^) (Table S2). In the third validation population, although the allelic association was not significant, significant genotypic association was detected between SNP rs28587458 and hypertension under an autosomal recessive model (*p_adj_* = 8.13 × 10^–4^, Table S4). Our finding that *FSTL5* is likely to be a possible susceptibility gene for hypertension is supported by the supplementary data in Table S7 in Lu et al^9^: SNP rs12512822 in intron nine of *FSTL5* and 37 kb from rs28587458 (r2 = 0.09, D’ = 0.31) showed suggestive association with blood pressure (*p *< 0.0001). *FSTL5* encodes a secretory glycoprotein of Follistatin Like 5 that inhibits the Wnt/β‐catenin signaling pathway,^10^ but its function in hypertension is not clear.

One major limitation of the current study is the size of the phase 1 discovery population was small. Therefore, the phase 1 GWAS population may be underpowered. However, this limitation may be attenuated by a multi‐stage study design with the discovery phase study followed by two consecutive validation and replication studies. Another limitation is that the functional studies were performed in endothelial cells in vitro, and the results may need to be validated in animal models or human patients in vivo.

In conclusion, our study identified two novel loci for hypertension, including SNP rs2064453 in the promoter of *GGT7* and SNP rs10847208 in lncRNA gene *LINC00943 LINC00944*. Our data also provide mechanistic insights into the genetic mechanism of hypertension and suggest that the risk allele T of rs2064453 increases risk of hypertension by increasing the expression of *GGT7*, which leads to reduced ERK1/2 activation and decreased expression of *PPP6C*.

## CONFLICT OF INTERESTS

The authors declare that they have no competing interests to declare

## ETHICAL APPROVAL

The studies were approved by the ethics committees of Huazhong University of Science and Technology, Shanghai Institute of Nutrition and Health (CAS) and Beijing Hospital. The studies conformed to the guidelines set forth by the Declaration of Helsinki, and written informed consent was obtained from the participants.

## FUNDING INFORMATION

This work was supported by the China National Natural Science Foundation grants (grant numbers: 81630002, 31671302, and 32070581).

## DATA AVAILABILITY STATEMENT

The data that support the findings of this study are openly available in the public Locuszoom (http://locuszoom.sph.umich.edu/) (accession number: 936641; Study ID: GeneID).

## Supporting information

SUPPORTING INFORMATION
