# Supplementary material for: A genome‐wide association study identifies novel association between genetic variants in GGT7 and LINC00944 and hypertension
Source: Clin Transl Med. 2021 May 21;11(5):e388. doi: 10.1002/ctm2.388 (PMC8140186; doi:10.1002/ctm2.388)
Supplement: Supplementary file 1 — SUPPORTING INFORMATION [file CTM2-11-e388-s001.docx]

**Supplementary Information**

**A genome-wide association study identifies novel association between genetic variants in *GGT7* and *LINC00944* and hypertension**

Chengcheng Tan^1,#^ | Hongfu Zhang^1,#^ | Dong Yu^1,#^ | Yao Hu^4,#^ | Pengxia Wang^1,#^ | Dan Wang^1,#^ | Jingjing Fa^1,#^ | Han Ran^1,#^ | Xiaoyu Zhang^1,#^ | Yanming Chen^2,#^ | Weixi Qin^1,5^| Chen Fang^1^ | Tie Ke^1^ | Nianguo Dong^3^ | Jianping Cai^6^ | Qing He^7^ | Shaofeng Huo^4^| Junhan Wang^2^ | Xiang Ren^1^ | Xin Tu^1^ | Xu Lin^4,*^ | Qing Wang^1,*^ | Chengqi Xu^1,*^

^1^Key Laboratory of Molecular Biophysics of the Ministry of Education, College of Life Science and Technology and Center for Human Genome Research, ^2^University-Affiliated Hospital, ^3^Department of Cardiovascular Surgery, Union Hospital, Tongji Medical College, Huazhong University of Science and Technology, Wuhan, P. R. China;

^4^CAS Key Laboratory of Nutrition, Metabolism and Food Safety, Shanghai Institute of Nutrition and Health, University of Chinese Academy of Sciences, Chinese Academy of Sciences, Shanghai, P. R. China;

^5^ Key Laboratory for Biorheological Science & Technology of Ministry of Education,

Bioengineering College of Chongqing University, Chongqing, P. R. China;

^6^Key Laboratory of Geriatrics, Ministry of Health, ^7^Department of Cardiology, Beijing Hospital, Beijing, 100730, P. R. China;

**Correspondence**

Dr. Chengqi Xu, College of Life Science and Technology and Human Genome Research Center, Huazhong University of Science and Technology, Wuhan, Hubei 430074, P. R. China; Tel/Fax: 086 027 8775 3502; Email: cqxu@mail.hust.edu.cn (C.X.); Dr. Xu Lin, CAS Key Laboratory of Nutrition, Metabolism and Food Safety, Shanghai Institute of Nutrition and Health, Chinese Academy of Sciences, Shanghai, P. R. China; Email: xlin@sibs.ac.cn (X.L.); Dr. Qing Wang, College of Life Science and Technology and Human Genome Research Center, Huazhong University of Science and Technology, Wuhan, Hubei 430074, P. R. China; Tel/Fax: 086 027 8775 3502; Email: qingwang118@qq.com (Q.W.);

^#^These authors contributed equally to this work.

**Materials and Methods**

**Study populations**

All study subjects were of Han ethnic origin. The phase 1 discovery population consisted of 353 patients affected with hypertension (cases) and 332 control study subjects without a diagnosis of hypertension randomly selected from the GeneID database. GeneID is a retrospective consecutive cohort from the Chinese Han population with >80,000 DNA samples and clinical data. The goal of the GeneID database is to identify susceptibility genes for cardiovascular and cerebrovascular diseases in the Chinese Han population. Using Gene ID database, we have identified a panel of genetic risk factors for coronary artery disease, stroke, atrial fibrillation and hypertension^1-9^. The phase 2 validation population consisted of 1,592 cases with hypertension and 1,302 controls without hypertension from the Nutrition and Health of Aging Population in China (NHAPC)^10^. NHAPC is a population-based cohort study with 3,289 individuals (50 to 70 years old) recruited from Beijing and Shanghai, and was used for GWAS of BMI, diabetes and hypertension as previously reported^11-14^. The phase 3 validation population consisted of 3,274 cases with hypertension and 2,734 controls without hypertension, also randomly selected from the GeneID database. Cases and controls in each study population were carefully evaluated, and matched by the geographical area of the birth and living to minimize sub-population stratification. To perform eQTL analysis, we enrolled 309 healthy study subjects without hypertension and undergoing annual physical examinations into GeneID. These 309 study subjects were not included into any of the case-control association studies.

The diagnosis of hypertension was made according to guidelines from the JACC Scientific Expert Panel^15^, the Scientific Statement from the American Heart Association^16^, and the guidelines from the JNC report^17^ and ESC/ESH^18^. Hypertension was defined as a constant, repeated systolic blood pressure (SBP) in the office of ≥140 mm Hg and/or diastolic blood pressure (DBP) ≥90 mm Hg, and lack of the secondary form of hypertension after extensive examinations that included tests of serum creatinine and electrolytes, urinalysis, and other hematologic screening. Patients were seated quietly for 5 minutes before office determination. Repetitive ambulatory blood pressure monitoring (ABPM) or home blood pressure monitoring for multiple days were used to confirm the definitive diagnosis of hypertension, and for diagnosis of white coat hypertension and masked hypertension. Study subjects undergoing anti-hypertensive treatments were also classified as hypertension cases. Controls were defined as individuals with SBP <140 mmHg, DBP <90 mmHg, and without antihypertensive treatments. Clinical data collected on the study subjects included age, gender, coronary artery disease, diabetes mellitus and the lipid profile (total cholesterol, LDL cholesterol, HDL cholesterol and triglyceride) if available from the medical files.

The studies were approved by the local ethics committees of the participating institutions, including Huazhong University of Science and Technology, Shanghai Institute of Nutrition and Health (CAS) and Beijing Hospital. The studies conformed to the guidelines set forth by the Declaration of Helsinki, and written informed consent was obtained from the participants. Whole blood was drawn from each participant, and genomic DNA was isolated from the blood samples using standard protocols^2^.

**Genome-wide genotyping and data processing**

Genotyping for GWAS was performed for the Discovery population with 353 patients with hypertension and 332 controls without hypertension. Genotyping was performed first for 266 cases and 189 controls using Affymetrix Genome-wide Human SNP5.0 arrays (Affymetrix, Santa Clara, CA) by commercial companies (Genetech, Shanghai and Capital Bio, Beijing) specialized in Affymetrix SNP array genotyping following standard experimental procedures from the manufacturer (Affymetrix). To increase the sample size, we performed genotyping for additional 87 cases and 143 controls using Affymetrix Genome-wide Human SNP6.0 arrays (Affymetrix, Santa Clara, CA) through Genetech Shanghai. Raw genotype calls were generated using Affymetrix Genotyping Console and then cleaned to generate high quality genotypes using a series of quality control (QC) procedures^2.^ The data were deposited to the public Locuszoom database (accession number: 936641, Study ID: GeneID). Subjects with low genotyping quality (calling rate <95%, contrast QC <0.4) were removed from further analysis. The genetic ancestry of GeneID study subjects were determined using principal component analysis (PCA) integrating 2,504 subjects from 5 populations from the 1000 Genome project, implemented in PLINK^19^.

For genotype imputation, genotypes of each set of GWAS data were first pre-phased at chromosomal levels by SHAPEIT^20^. Untyped genotypes were imputed using a merged reference panel from 1000 Genomes Phase 3 data across all 2,504 samples as reference haplotype data by IMPUTE2^21^ with 4 MB per each chunk. The other parameters were default parameters in IMPUTE2. Imputed genotypes with low imputation quality (Info<0.4) were excluded from further analysis. We then took the intersections of two sets of the imputed data and further filtered out variants with a minor allele frequency (MAF) of <0.05, missing genotyping rate of >0.1 and *P* values of <1×10^-3^ from Hardy-Weinberg equilibrium tests. After quality control, we obtained 3,956,088 autosomal SNPs for subsequent association analysis.

***In silico* replication of selected positive variants from GWAS in the NHAPC population**

The NHAPC population with 1,592 patients with hypertension and 1,302 controls without hypertension was used as the phase 2 replication population for this study. Genotyping for the NHAPC population was performed using the Illumina Human660W-Quad BeadChip (Illumina, Inc., San Diego, CA) by Bio-X Center and Chinese National Human Genome Center in Shanghai as previously reported^11, 14^. SNPs were filtered out if they had a call rate of <95%, a MAF of 0–5%, and *P* values of 10^-6^ from Hardy-Weinberg equilibrium tests in the control group. A total of 495,686 variants were obtained for further analysis. Imputation was performed for the genotyping data from the NHAPC population as described previously^11, 14^. The genotyping data from 1,592 patients with hypertension and 1,302 controls without hypertension from NHAPC were then analyzed for association of selected SNPs showing positive association with hypertension in the Discovery population.

**Genotyping of the phase 3 validation population**

Our third stage validation population consisted of 3,274 cases with hypertension and 2,734 controls without hypertension from GeneID. Genotyping of five lead SNPs in the validation population was carried out using the High Resolution Melt (HRM) analysis, and nine lead SNPs were genotyped using TaqMan genotyping assays. HRM analysis was carried out using a Rotor-GeneTM 6000 High Resolution Melt (HRM) system, and TaqMan genotyping was carried out using the QuantStudio™ 12K Flex Real-Time PCR System as described previously^1, 2^. **Table S6** lists the primers used for HRM analysis and TaqMan Assays-on-Demand ID numbers for TaqMan genotyping. The original genotyping data were called independently by two researchers in a blind manner. When conflicting genotype calls occurred, Sanger sequencing was used for final verification. Sanger DNA sequence analysis was performed using the BigDye Terminator v3.1 Cycle Sequencing Kit (Thermo Fisher Scientific, Waltham, MA, USA).

**Association analysis**

Case-control association analysis for hypertension was performed with logistic regression analysis with adjustment for covariates of sex, age, and age^2^ as reported for common complex cardiovascular diseases^22-24^ as well as three leading principal components using PLINK as described^19^. Quantile-quantile plots and principle component analysis (PCA) were carried out for the discovery population using the R package (version 3.0.1). We used PLINK to calculate the genomic control lambda values for the discovery population. At the validation stage, association of SNPs with hypertension was assessed using Pearson’s 2×2 and 2x3 contingency table χ2 tests or Fisher’s exact tests (SPSS Statistics, version 17.0 or PLINK version 1.07) when the value in one of the four cells of a contingency table is below 5. Odds ratios (ORs) and 95% confidence intervals (CIs) were estimated using the χ2 test (SPSS Statistics, version 17.0) as described previously^1, 2^. Multivariate regression analysis was performed by incorporating age, age^2^ and sex as covariates with logistic regression analysis (SPSS Statistics, version 17.0) as described previously^1, 2, 22-24^. SNPs were tested for Hardy-Weinberg equilibrium in control populations using the Haploview software package version 3.0. The heterogeneity between two cohorts was further evaluated for each variant using a Cochran’s Q test implemented in statistical program GWAMA^25^. If significant heterogeneity (pHet<0.05 and I^2^>70%) was identified, pooled results from the random effect model (assuming a heterogeneous genetic effect) were reported.

For the phase 1 GWAS in the Discovery GeneID population, we used a conservative *P* value of <5x10^-3^ as the cutoff threshold to select as many positive SNPs as possible for further replication (i.e. to minimize false negatives). The same rationale and cutoff threshold *P*<5x10^-3^ were applied to the phase 2 *in silico* replication study using the NHAPC population. For the phase 3 validation study using the GeneID population, we tested 14 SNPs, and used the most stringent Bonferroni correction for adjusting for multiple testing (*P*<0.05/14 =3.57x10^-3^).

**Expression quantitative trait loci (eQTL) assays and real-time RT-PCR analysis**

Total RNA samples were extracted from peripheral blood leukocytes from 309 healthy individuals enrolled into GeneID and from cultured EA.hy926 endothelial cells using the TRIzol reagent according to the manufacturer’s instruction (Thermo Fisher Scientific, Waltham, MA, USA). One microgram of total RNA was used for reverse transcription to generate cDNA with HiScript® II Q Select RT SuperMix for qPCR (+gDNA wiper) (Vazyme, Nanjing, China). The cDNA was used for real time PCR assays with the AceQ® Universal SYBR qPCR Master Mix (Vazyme, Nanjing, China) as described ^26^. The internal reference genes were *ACTB* encoding β-actin for eQTL analysis and *GAPDH* for EA.hy926 studies.

The comparative Cт ($\Delta\Delta$Cт) analysis was performed to measure the relative expression level of mRNA on the QuantStudio™ 12K Flex Real-Time PCR System (Applied Biosystems). The raw data were quality-controlled by QuantStudio™ 12K Flex software v1.3. The outliers according to the QC summary were excluded, and the software output generated the relative quantification values, which were used for further analysis. Gene expression data from our qPCR experiments were determined by relative quantification (RQ) defined as 2^-∆∆Cт^. The ∆∆Cт value represented the difference of PCR cycles of a target gene between two groups calibrated by an internal control gene. We first used log2 transformation to convert RQ values, and then employed inverse normal transformation (INT) to ensure normality of residuals before eQTL analysis.

Genomic DNA was also extracted from whole blood samples from the 309 individuals using standard protocols as reported previously^2^. SNPs rs2064453 and rs10847208 were genotyped using the HRM analysis as described above. The primers used for HRM analysis are shown in **Table S6**. eQTL analysis was performed by correlating the expression levels of candidate genes and genotypes of the two SNPs, and adjusted by age and gender using a linear regression model using SPSS version.17.0.

**Identification of *GGT7*-linked genes using HumanBase**

Using the online tool of the web server for HumanBase (data-driven predictions of gene expression, function, regulation, and interactions in human) (https://hb.flatironinstitute.org/)^27-30^, we identified genes that are linked with *GGT7*. We examined 11 tissues involved in the development of hypertension, including the adrenal cortex, intestine, kidney, liver, nephron, renal tubule, serum, smooth muscle, and endothelial cells. Parameters include: (1) predicted to interact with *GGT7* in at least two tissues, (2) the score of the average weight of connections of above 0.5, and (3) related to eight or more aforementioned 11 tissues even without association with blood pressure regulation.

**Plasmids and siRNAs**

The promoter and regulatory region of the *GGT7* gene starting from -1,967 bp to +72 bp from the transcriptional start site (TSS) was amplified by PCR analysis using human genomic DNA as the template. The PCR primers include GGT7-promoter-kpnI-F (5’-catttctctatcgataggtaccTACAGACCTCTTGGGGTGGACATAGACC-3’) and GGT7-promoter-hindIII-R (5’-cagtaccggaatgccaagcttCCTGGCTGGCCTCGTTCTCCGC-3’). The PCR product was subcloned into the pGL3-basic luciferase vector by homologous recombination (TaKaRa, Dalian, China). The subcloning was performed with incubation at 37℃ for 30 min using the ClonExpress® II One Step Cloning Ki (Vazyme, Nanjing, China). This generates the wild type luciferase reporter for the *GGT7* promoter, referred to as pGL3-Basic-rs2064453-T.

Site-directed mutagenesis was performed for pGL3-Basic-rs2064453-T to introduce the minor allele C of SNP rs2064453 as described^31^, resulting in the mutant luciferase reporter pGL3-Basic-rs2064453-C.

A similar strategy was used to create two luciferase reporters for SNP rs10847208, the pGL3-promoter-rs10847208-C to assess the effect of the variant on a potential enhancer and the pMIR-REPORT-rs10847208-C to assess the potential effect of the variant on miRNA binding and RNA stability. We amplified a 201 bp DNA fragment spanning the SNP using human genomic DNA as the template as above, and sub-cloned it into the pGL3-promoter vector (Promega, Madison, Wisconsin, USA) and the pMIR-REPORT vector (Applied Biosystems, Foster City, CA, USA). Site-directed mutagenesis was used to create mutant reports with the minor allele T of SNP rs10847208, pGL3-promoter-rs10847208-T and the pMIR-REPORT-rs10847208-T.

The full-length cDNA for *GGT7* (UniProt entry: Q9UJ14-1; HGNC: 4259) was ampliﬁed as several short fragments by RT-PCR analysis using RNA samples from HEK293 cells. Homologous recombination was used to join the short fragments together using the ClonExpress® II One Step Cloning Kit (Vazyme, Nanjing, China). The PCR product was sub-cloned into the pET-28a(+) vector for *E. coli* expression and purification of 6xHis-tagged GGT7 protein (pET28-GGT7).

We purchased siRNA for the *GGT7* gene from Genepharma (Suzhou, China).

GGT7-si: 5’-GGACCAACAACUUCAUCAUTT-3’ (sense) and 5’-AUGAUGAAGUUGUUGGUCCTT-3’ (antisense).

NC-si: 5’-UUCUCCGAACGUGUCACGUTT-3’ (sense) and 5’-ACGUGACACGUUCGGAGAATT-3’ (antisense).

**Cell culture and transfection**

EA.hy926 endothelial cells were purchased from ATCC (American Type Culture Collection, Manassas, VA, USA, ATCC CRL-2922) and maintained in human endothelial basal growth media supplemented with 10% fetal bovine serum (FBS) (Gibco Life Technologies, Gaithersburg, MD, USA). HEK293, a human embryonic kidney cell line (ATCC CRL-1573), was cultured in DMEM with 10% FBS. All cells were cultured at 5% CO_2_ and 37°C in a humidiﬁed cell culture incubator. Transfection of siRNA was performed for Eahy926 cells using the Fu GENE® HD Transfection Reagent (Promega, Madison, Wisconsin, USA) according to the manufacturer's instruction. Plasmid DNA was transfected into HEK293 cells using Polyethyleneimine^32^ with a ratio of ¾ for PEI buffer (5 uM, pH 7.4) over HBS buffer (8.76 g NaCl, 20 ml 1 M HEPES in 1L dH2O, pH 7.4) in 6-well culture plates. Two micrograms of plasmid DNA was mixed with 75 ul of PEI buffer and 100 ul of HBS buffer, and incubated for 20 minutes at room temperature before adding into the culture wells.

**Dual-luciferase assays**

HEK293 cells were cultured in 24-well plates. After 24 hours, each well was transfected with 250 ng of a luciferase reporter and 20 ng of the pRL-TK vector containing the renilla luciferase gene (Promega, Madison, WI, USA). Cells were harvested 48 hours after transfection and used for dual luciferase assays using the Dual-luciferase reporter assay system (Promega, Madison, Wisconsin, USA) according to the manufacturer's instructions. The ﬁreﬂy luciferase activity was normalized to the corresponding Renilla luciferase activity as described ^33^.

**Protein purification**

The pET-28-GGT7 expression plasmid was transformed into *Escherichia coli* BL21 (DE3) Star. Expression of the 6xHis-tagged GGT7 protein was induced using IPTG, and purified using a Ni-NTA agarose column according to the manufacturer's instructions (Qiagen, Dusseldorf, Germany) and as reported^34^. The purified protein was dialyzed, and its quality was examined by SDS-PAGE and Coomassie blue staining as well as Western blot analysis. The purified 6xHis–GGT7 protein was treated with desalinization (Zeba Spin Desalting Columns, 89891, Thermo Fisher Scientiﬁc, Waltham, MA, USA) before use.

**Western blot analysis**

Western blot analysis was carried out as described by us previously ^35^. Cells were lysed with Western blot cell lysis buffer (Beyotime, Shanghai, China) with a cocktail of protease inhibitors (Roche, Mannheim, Germany). The cell extracts were separated by 12% SDS-PAGE and transferred to nitrocellulose membranes. The membranes were blocked with 5% skim milk for 2 hours and incubated with primary antibodies overnight at 4°C. The primary antibodies used in the study include a rabbit antibody against GGT7 (1:1000 dilution; 24674-1-AP, Proteintech, Wuhan, China), a mouse monoclonal antibody (mAb) against Alpha Tubulin (1:5000 dilution; 66031-1-Ig, Proteintech, Wuhan, China) and a rabbit mAb against total ERK1/2 and phosphorylated ERK1/2 (Thr202/Tyr204) (1:3000 dilution; #9102 and #4370, Cell Signaling Technology, Danvers, MA, USA). The secondary antibodies included a goat anti-rabbit antibody and a goat anti-mouse antibody (1:10,000 dilution; #W10809 and #W10808, Thermo Fisher Scientiﬁc, Waltham, MA, USA).

**Other statistical analysis**

For analysis of quantitative data, a two-sided Student's *t* test was used for analysis of two groups of samples and one-way ANOVA for analysis of more than two groups of samples using SPSS version 17.0.

**REFERENCES**

1. Chen S, Wang X, Wang J, et al. Genomic variant in CAV1 increases susceptibility to coronary artery disease and myocardial infarction. *Atherosclerosis*. 2016;246:148-156.
2. Wang F, Xu CQ, He Q, et al. Genome-wide association identifies a susceptibility locus for coronary artery disease in the Chinese Han population. *Nature Genet*. 2011;43(4):345-349.
3. Xu C, Wang F, Wang B, et al. Minor allele C of chromosome 1p32 single nucleotide polymorphism rs11206510 confers risk of ischemic stroke in the Chinese Han population. *Stroke*. 2010;41(8):1587-1592.
4. Yang Q, Xiong H, Xu C, et al. Identification of rare variants in cardiac sodium channel β4-subunit gene SCN4B associated with ventricular tachycardia. *Mol Genet Genomics*. 2019;294(4):1059-1071.
5. Huang Y, Wang C, Yao Y, et al. Molecular Basis of Gene-Gene Interaction: Cyclic Cross-Regulation of Gene Expression and Post-GWAS Gene-Gene Interaction Involved in Atrial Fibrillation. *PLoS Genet*. 2015;11(8):e1005393.
6. Tu X, Nie S, Liao Y, et al. The IL-33-ST2L pathway is associated with coronary artery disease in a Chinese Han population. *Am J Hum Genet*. 2013;93(4):652-660.
7. Shi L, Li C, Wang C, et al. Assessment of association of rs2200733 on chromosome 4q25 with atrial fibrillation and ischemic stroke in a Chinese Han population. *Hum Genet*. 2009;126(6):843-849.
8. Cheng X, Shi L, Nie S, et al. The same chromosome 9p21.3 locus is associated with type 2 diabetes and coronary artery disease in a Chinese Han population. *Diabetes*. 2011;60(2):680-684.
9. Xu C, Yang Q, Xiong H, et al. Candidate pathway-based genome-wide association studies identify novel associations of genomic variants in the complement system associated with coronary artery disease. *Circ-Cardiovasc Genet*. 2014;7(6):887-894.
10. Ye X, Yu Z, Li H, Franco OH, Liu Y, Lin X. Distributions of C-reactive protein and its association with metabolic syndrome in middle-aged and older Chinese people. *J Am Coll Cardiol*. 2007;49(17):1798-1805.
11. Wen W, Zheng W, Okada Y, et al. Meta-analysis of genome-wide association studies in East Asian-ancestry populations identifies four new loci for body mass index. *Hum Mol Genet*. 2014;23(20):5492-5504.
12. Lu X, Wang L, Lin X, et al. Genome-wide association study in Chinese identifies novel loci for blood pressure and hypertension. *Hum Mol Genet*. 2015;24(3):865-874.
13. Kato N, Loh M, Takeuchi F, et al. Trans-ancestry genome-wide association study identifies 12 genetic loci influencing blood pressure and implicates a role for DNA methylation. *Nature Genet*. 2015;47(11):1282-1293.
14. Li H, Gan W, Lu L, et al. A genome-wide association study identifies GRK5 and RASGRP1 as type 2 diabetes loci in Chinese Hans. *Diabetes*. 2013;62(1):291-298.
15. Muntner P, Einhorn PT, Cushman WC, et al. Blood Pressure Assessment in Adults in Clinical Practice and Clinic-Based Research: JACC Scientific Expert Panel. *J Am Coll Cardiol*. 2019;73(3):317-335.
16. Muntner P, Shimbo D, Carey RM, et al. Measurement of Blood Pressure in Humans: A Scientific Statement From the American Heart Association. *Hypertension*. 2019;73(5):e35-e66.
17. Chobanian AV, Bakris GL, Black HR, et al. The Seventh Report of the Joint National Committee on Prevention, Detection, Evaluation, and Treatment of High Blood Pressure: the JNC 7 report. *JAMA-J Am Med Assoc*. 2003;289(19):2560-2572.
18. Williams B, Mancia G, Spiering W, et al. 2018 ESC/ESH Guidelines for the management of arterial hypertension: The Task Force for the management of arterial hypertension of the European Society of Cardiology and the European Society of Hypertension: The Task Force for the management of arterial hypertension of the European Society of Cardiology and the European Society of Hypertension. *J Hypertens*. 2018;36(10):1953-2041.
19. Purcell S, Neale B, Todd-Brown K, et al. PLINK: a tool set for whole-genome association and population-based linkage analyses. *Am J Hum Genet*. 2007;81(3):559-575.
20. Delaneau O, Marchini J, Zagury JF. A linear complexity phasing method for thousands of genomes. *Nat Methods*. 2011;9(2):179-181.
21. Howie BN, Donnelly P, Marchini J. A flexible and accurate genotype imputation method for the next generation of genome-wide association studies. *PLoS Genet*. 2009;5(6):e1000529.
22. Liu C, Kraja AT, Smith JA. Meta-analysis identifies common and rare variants influencing blood pressure and overlapping with metabolic trait loci. *Nature Genet*. 2016;48(10):1162-1170.
23. Padmanabhan S, Melander O, Johnson T, et al. Genome-wide association study of blood pressure extremes identifies variant near UMOD associated with hypertension. *PLoS Genet*. 2010;6(10):e1001177.
24. Giri A, Hellwege JN, Keaton JM, et al. Trans-ethnic association study of blood pressure determinants in over 750,000 individuals. *Nature Genet*. 2019;51(1):51-62.
25. Magi R, Morris AP. GWAMA: software for genome-wide association meta-analysis. *BMC bioinformatics*. 2010;11:288.
26. Bai Y, Zhang Y, Han B, et al. Circular RNA DLGAP4 Ameliorates Ischemic Stroke Outcomes by Targeting miR-143 to Regulate Endothelial-Mesenchymal Transition Associated with Blood-Brain Barrier Integrity. *J Neurosci*. 2018;38(1):32-50.
27. Greene CS, Krishnan A, Wong AK, et al. Understanding multicellular function and disease with human tissue-specific networks. *Nature Genet*. 2015;47(6):569-576.
28. Krishnan A, Zhang R, Yao V, et al. Genome-wide prediction and functional characterization of the genetic basis of autism spectrum disorder. *Nat Neurosci*. 2016;19(11):1454-1462.
29. Zhou J, Theesfeld CL, Yao K, Chen KM, Wong AK, Troyanskaya OG. Deep learning sequence-based ab initio prediction of variant effects on expression and disease risk. *Nature Genet*. 2018;50(8):1171-1179.
30. Zhou J, Troyanskaya OG. Predicting effects of noncoding variants with deep learning-based sequence model. *Nat Methods*. 2015;12(10):931-934.
31. Zhao Y, Huang Y, Li W, et al. Post-transcriptional regulation of cardiac sodium channel gene SCN5A expression and function by miR-192-5p. *Biochim Biophys Acta-Mol Basis Dis*. 2015;1852(10 Pt A):2024-2034.
32. Yang S, Zhou X, Li R, Fu X, Sun P. Optimized PEI-based Transfection Method for Transient Transfection and Lentiviral Production. *Curr Protoc Chem Biol*. 2017;9(3):147-157.
33. Si W, Xie W, Deng W, et al. Angiotensin II increases angiogenesis by NF-kappaB-mediated transcriptional activation of angiogenic factor AGGF1. *Faseb J*. 2018;32(9):5051-5062.
34. Lu Q, Yao Y, Hu Z, et al. Angiogenic Factor AGGF1 Activates Autophagy with an Essential Role in Therapeutic Angiogenesis for Heart Disease. *PLoS Biol*. 2016;14(8):e1002529.
35. Yao Y, Lu Q, Hu Z, Yu Y, Chen Q, Wang QK. A non-canonical pathway regulates ER stress signaling and blocks ER stress-induced apoptosis and heart failure. *Nat Commun*. 2017;8(1):133.


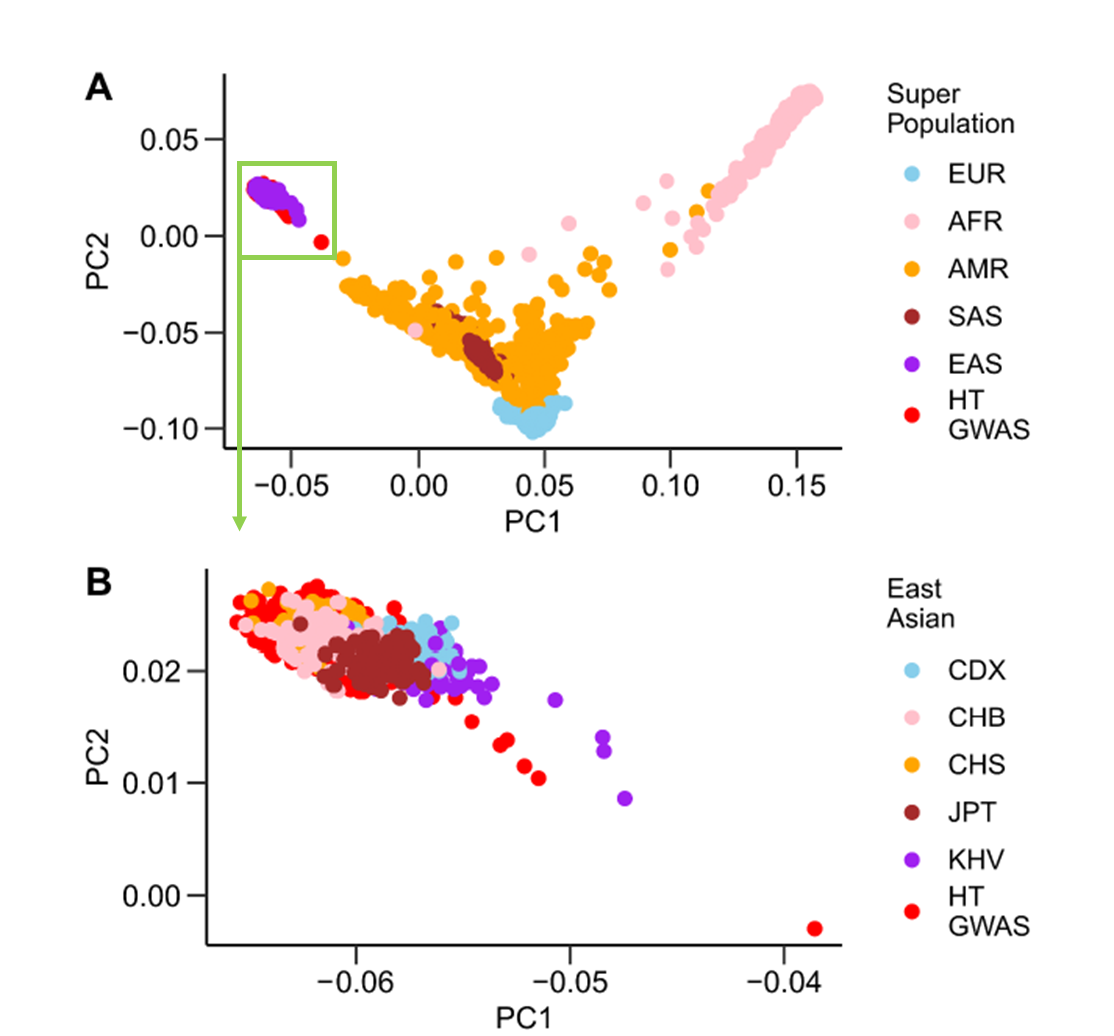


**Figure S1. Population structure of the study subjects at the discovery stage (GeneID-I). (A)** Scatter plot showing genetic distance between 685 samples from GeneID-I (red, HT-GWAS) and 5 super-populations in the phase 3 1000 Genome project. The first two principal components are calculated by PCA. EUR, Europeansl; AFR, African Americans; AMR, Ad Mixed Americans; SAS, South Asians; EAS, East Asians. **(B)** Zoomed-in plot showing the GWAS study subjects (HT-GWAS) together with five sub-populations of East Asian ancestry. The principal component analysis (PCA) showed that the GWAS samples were consistent with East Asian samples in 1000Genome project. CDX, Chinese Dai in Xishuangbanna; CHB, Han Chinese in Beijing; CHS, Southern Han Chinese; JPT, Japanese in Tokyo; KHV, Kinh in Ho Chi Minh City.

**
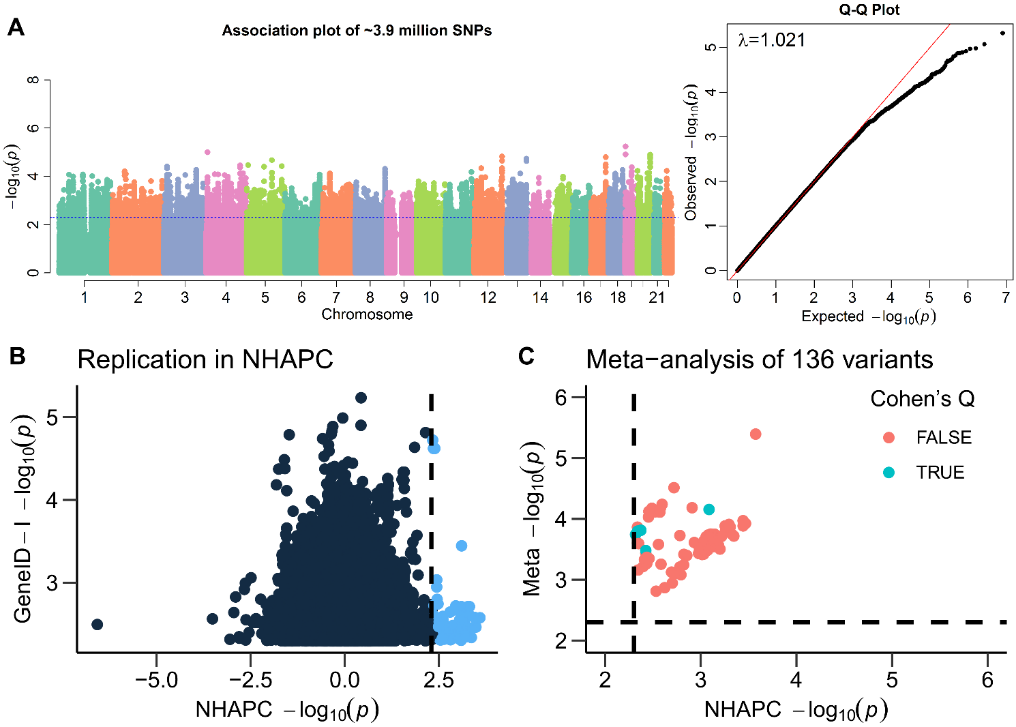
**

**Figure S2. Genetic association analyses in the discovery and replication samples.** (A) Manhattan and Q-Q plots showing genome-wide association results in the discovery population (GeneID-I). After quality control, 3,956,088 SNPs were analyzed for their association with hypertension with adjustment of gender, age, age^2 and the first three principal components. (B). Replication of 17,435 SNPs in the replication population (NHAPC). Among 17,435 SNPs from phase 1, 136 SNPs showed *P* values of less than 5.0 x 10^-3^ and concordant direction of effect, and clustered into 15 independent loci. (C). Meta-analysis of 136 variants showing *P*<0.005 in both GeneID-I and NHAPC populations. Most of 136 SNPs showed no significant heterogeneity between the two cohorts. Colors indicate heterogeneity of genetic effects between two populations.

**
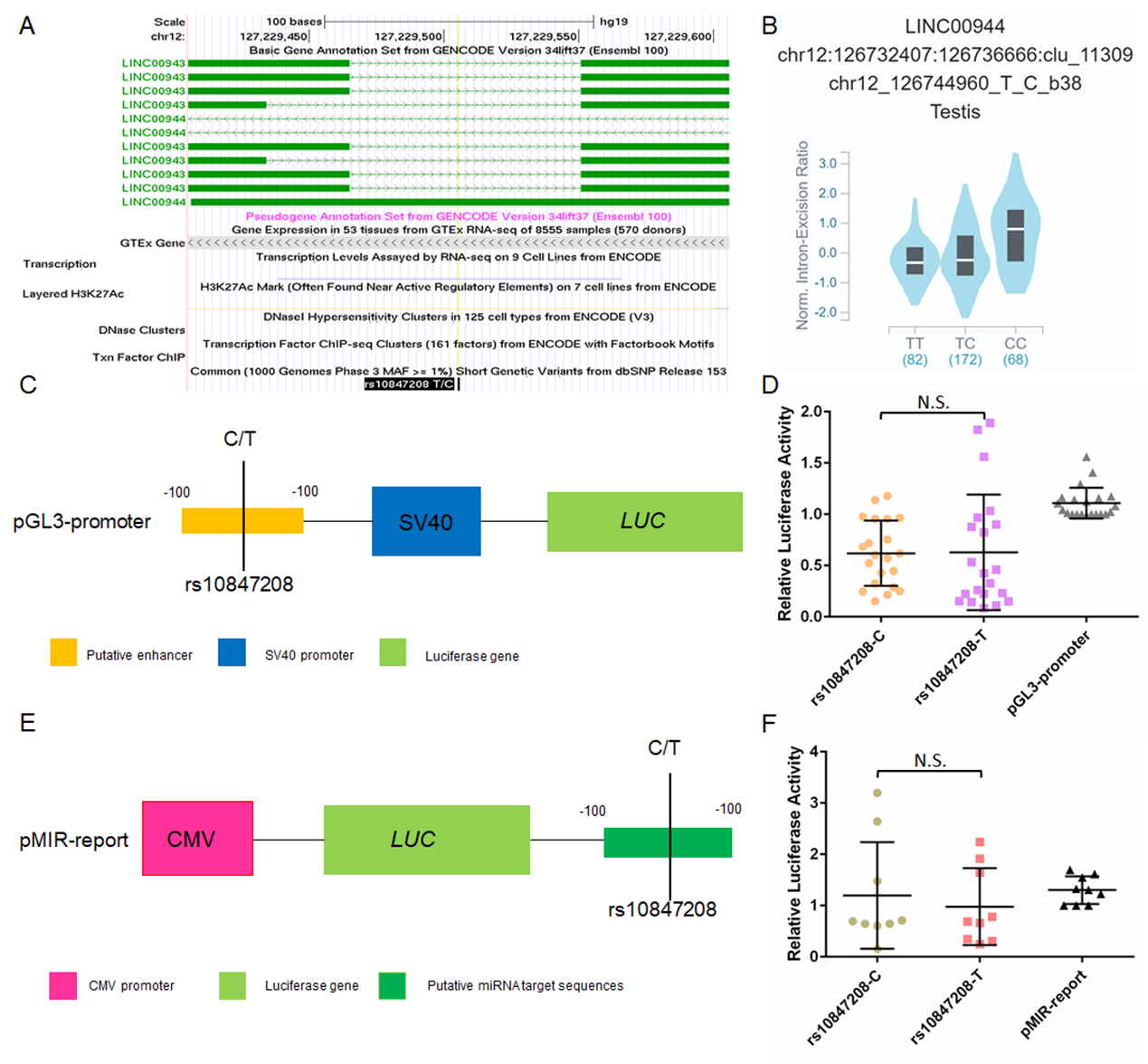
**

**Figure S3. Identification of eQTL between the lead variant rs10847208 at the new hypertension locus on chromosome 12q24.32 and lncRNA gene *LINC00944.***

**A:** Genomic position of SNP rs10847208 (http://genome.ucsc.edu/cgi-bin/hgTrackUi?db=hg19&g= wgEncodeRegTfbsClusteredV3).  **B：** SNP rs10847208 is an eQTL for lncRNA gene *LINC00944* in testes with *P*=7.8 x 10^-7^. **C:** Luciferase reporters pGL3-promoter-rs10847208-C and pGL3-promoter-rs10847208-T for assessing the effect of SNP rs10847208 on transcription activation (e.g. an enhancer function).  **D:** Luciferase assays showing no significant differences between risk allele C and allele T of SNP rs10847208 on transcription activation(n=21).  **E:** Luciferase reporters and pMIR-REPORT-rs10847208-C and pMIR-REPORT-rs10847208-T for assessing the effect of SNP rs10847208 on RNA stability and translation (e.g. a microRNA binding site and function).  **F：** Luciferase assays showing no significant differences between risk allele C and allele T of SNP rs10847208 on RNA stability and translation(n=9). N.S., no significant, *P*>0.05. Line is for mean with SD (standard deviation). P value was obtained by Student's t test.

**
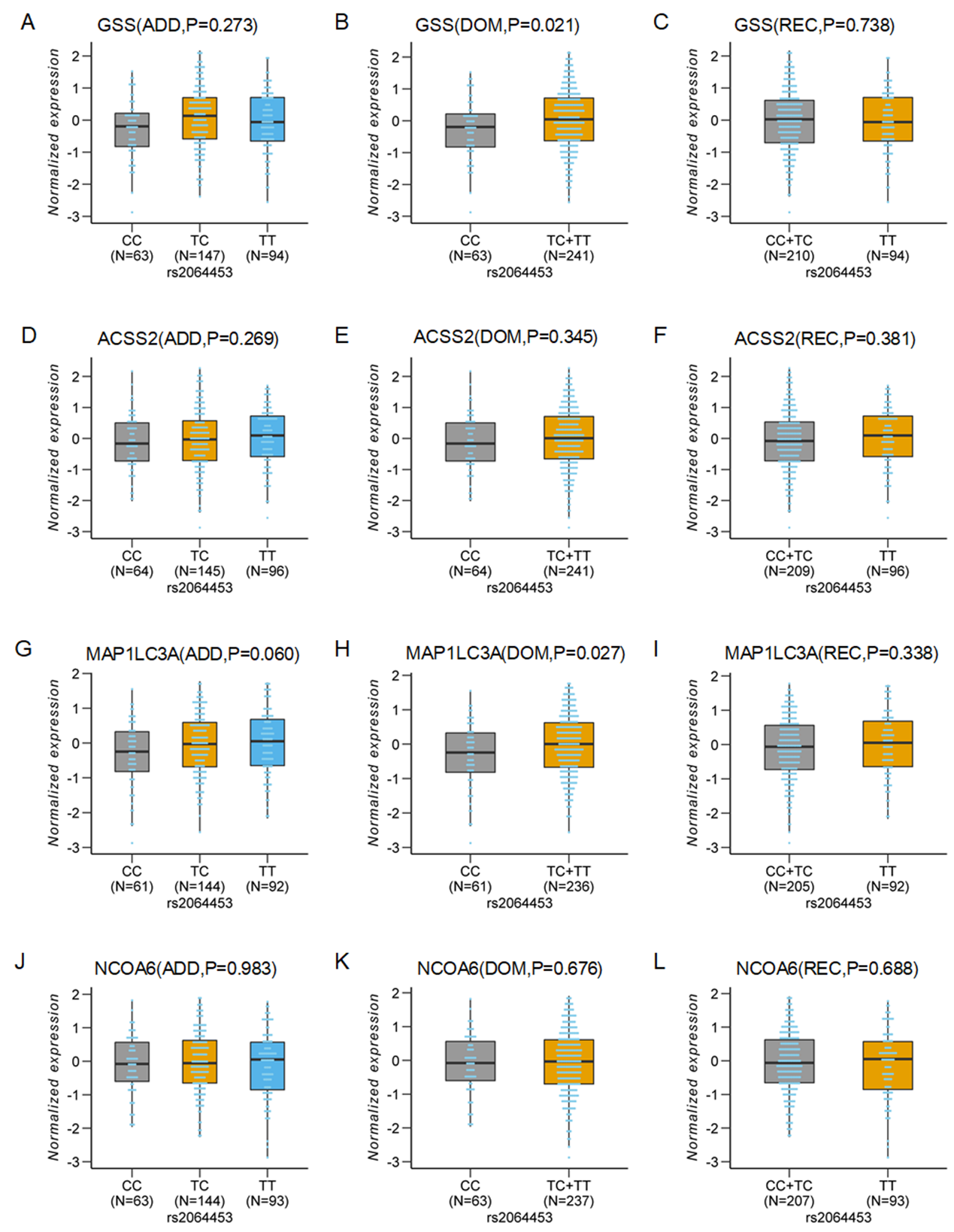
**

**Figure S4. eQTL analysis between the lead variant rs2064453 at the new hypertension locus on chromosome 20q11.22 and nearby genes under an additive (ADD), dominant (DOM), or recessive model (REC).** (**A-C**) eQTL analysis for *GSS*. (**D-F**) eQTL analysis for *ACSS2.* (**G-I**) eQTL analysis for *MAP1LC3A*. (**J-K**) eQTL analysis for *NCOA6*. *P* values are indicated at the top of each plot. The number of study subjects is indicated with N.

P value was obtained by linear regression after adjustment with age and gender.

| **Table S1. Demographical and clinical characteristics of the discovery, validation and replication populations for GWAS in the Chinese Han population** | | | | | | | | | |
| --- | --- | --- | --- | --- | --- | --- | --- | --- | --- |
|  |  | Age | Gender | CAD | DM | Tch | TG | HDL-c | LDL-c |
| Population | | (years) | Female *n*（%) | *n* (%) | *n* (%) | mmol/L | mmol/L | mmol/L | mmol/L |
| Discovery (Stage 1) | |  |  |  |  |  |  |  |  |
| AFFY5 |  |  |  |  |  |  |  |  |  |
|  | Cases (*n=*266) | 60.84±8.99 | 131(49.2) | 135(51.0) | 39(14.7) | 4.58±1.01 | 1.80±1.26 | 1.16±0.28 | 2.69±0.75 |
|  | Controls (*n=*189) | 59.61±8.62 | 76(40.2) | 92(48.7) | 22(11.6) | 4.46±1.14 | 1.78±1.05 | 1.19±0.46 | 2.61±0.79 |
|  | *P* | 0.145 | 0.056 | 0.663 | 0.554 | 0.248 | 0.898 | 0.396 | 0.276 |
| AFFY6 |  |  |  |  |  |  |  |  |  |
|  | Cases (*n=*87) | 64.01±9.95 | 39(44.8) | 14 (16.1) | 13(14.9) | 4.50±1.07 | 1.40±0.80 | 1.18±0.33 | 2.73±0.90 |
|  | Controls (*n=*143) | 57.87±12.70 | 45(31.5) | 7 (4.9) | 7(4.9) | 4.68±1.03 | 1.63±1.17 | 1.19±0.31 | 2.61±0.76 |
|  | *P* | 0.0002 | 0.041 | 0.005 | 0.009 | 0.509 | 0.382 | 0.901 | 0.603 |
| Combined |  |  |  |  |  |  |  |  |  |
|  | Cases(*n*=353) | 61.62±9.32 | 170(48.2) | 149(42.2) | 52(14.7) | 4.58±1.02 | 1.76±1.07 | 1.17±0.28 | 2.69±0.76 |
|  | Controls(*n*=332) | 58.86±10.56 | 121(36.4) | 99(29.8) | 29(8.7) | 4.50±1.13 | 1.76±1.23 | 1.19±0.44 | 2.61±0.79 |
|  | *P* | 0.0003 | 0.002 | 0.0008 | 0.005 | 0.434 | 0.979 | 0.383 | 0.233 |
| Replication (Stage 2) | |  |  |  |  |  |  |  |  |
| NHAPC |  |  |  |  |  |  |  |  |  |
|  | Cases (*n=*1592) | 58.67±6.03 | 898(56.4) | NA | 240(15.1) | 4.72±1.01 | 1.41±1.12 | 1.28±0.33 | 3.28±0.99 |
|  | Controls (*n=*1302) | 58.60±5.99 | 745(57.2) | NA | 149(11.4) | 4.68±0.92 | 1.38±1.01 | 1.27±0.33 | 3.26±0.94 |
|  | *P* | 0.755 | 0.661 | NA | 0.004 | 0.270 | 0.454 | 0.417 | 0.580 |
| Validation (Stages 3) | |  |  |  |  |  |  |  |  |
| GeneID |  |  |  |  |  |  |  |  |  |
|  | Cases (*n=*3274) | 62.7±13.57 | 1337(40.8) | 236 (7.2) | 769(23.5) | 4.78±1.20 | 1.76±1.58 | 1.19±0.46 | 2.84±0.91 |
|  | Controls (*n=*2734) | 48.1±12.40 | 1184(43.3) | 1 (0.03) | 123(4.5) | 5.15±0.94 | 1.54±1.40 | 1.46±0.40 | 3.17±0.84 |
|  | *P* | <0.0001 | 0.053 | <0.0001 | <0.0001 | <0.0001 | 0.004 | <0.0001 | <0.0001 |
| Age for cases, age at the first diagnosis; Age for controls, age at the time of enrollment. CAD, coronary artery disease; Tch, total cholesterol; TG, triglyceride; HDL-c, high-density lipoprotein cholesterol; LDL-c, low-density lipoprotein cholesterol; NA, no data available. Data for the age, Tch, TG, HDL-c, and LDL-c are shown as mean ± standard deviation.  P value for Gender, CAD and DM were obtained by Chi-squared Test, P value for Age, Tch, TG, HDL-c and LDL-c were obtained by Student’s t Test. | | | | | | | | | |

| **Table S2. Identification of 136 variants showing association with hypertension with *P*<0.005 in both discovery and replication populations** | | | | | | | | | | | | | | | | | | | | | | | |
| --- | --- | --- | --- | --- | --- | --- | --- | --- | --- | --- | --- | --- | --- | --- | --- | --- | --- | --- | --- | --- | --- | --- | --- |
| **Lead** | **SNP** | **CHR** | **Position** | **Function** | **Gene** | **1KG_EAS** | **EA** | **NEA** | **GeneID-I (N=685)** | | |  | **NHAPC (N=2894)** | | |  | **Meta-analysis of GeneID-I and NHAPC (N=3579)** | | | | | | |
|  |  |  |  |  |  |  |  |  | **EAF** | **OR (95%CI)** | ***P*** |  | **EAF** | **OR (95%CI)** | ***P*** |  | **EAF** | **OR (95%CI)** | ***P*fixed** | ***P*random** | **Q** | **I2** | **Direction** |
| 0 | rs12402358 | 1 | 108393348 | intronic | *VAV3* | 0.123 | T | A | 0.890 | 1.69(1.18-2.42) | 4.5E-03 |  | 0.900 | 1.24(1.05-1.47) | 3.8E-03 |  | 0.898 | 1.31(1.13-1.53) | 5.3E-04 | 2.7E-02 | 0.128 | 0.568 | ++ |
| 1 | rs7517496 | 1 | 108393995 | intronic | *VAV3* | 0.125 | T | C | 0.889 | 1.70(1.19-2.44) | 3.9E-03 |  | 0.900 | 1.24(1.05-1.47) | 4.0E-03 |  | 0.898 | 1.32(1.13-1.54) | 4.6E-04 | 2.9E-02 | 0.119 | 0.589 | ++ |
| 1 | rs6436354 | 2 | 157231579 | intergenic | *NR4A2,GPD2* | 0.224 | G | A | 0.828 | 1.53(1.14-2.04) | 4.5E-03 |  | 0.806 | 1.23(1.08-1.40) | 1.3E-03 |  | 0.810 | 1.27(1.13-1.44) | 6.4E-05 | 6.6E-03 | 0.180 | 0.445 | ++ |
| 1 | rs6756964 | 2 | 158645538 | intronic | *ACVR1* | 0.205 | G | A | 0.247 | 1.50(1.16-1.95) | 2.5E-03 |  | 0.215 | 1.16(1.03-1.32) | 2.7E-03 |  | 0.222 | 1.22(1.09-1.37) | 5.5E-04 | 4.1E-02 | 0.087 | 0.658 | ++ |
| 1 | rs3774268 | 3 | 186954324 | Synonymous | *MASP1* | 0.132 | A | G | 0.094 | 1.83(1.25-2.68) | 2.2E-03 |  | 0.079 | 1.30(1.07-1.58) | 1.7E-03 |  | 0.082 | 1.40(1.17-1.66) | 1.8E-04 | 1.8E-02 | 0.121 | 0.583 | ++ |
| 0 | rs12642902 | 4 | 123508501 | intergenic | *IL2,IL21* | 0.484 | A | G | 0.488 | 1.40(1.11-1.77) | 4.4E-03 |  | 0.477 | 1.17(1.05-1.29) | 4.7E-03 |  | 0.479 | 1.20(1.09-1.32) | 1.3E-04 | 1.4E-02 | 0.151 | 0.515 | ++ |
| 0 | rs6835457 | 4 | 123511126 | intergenic | *IL2,IL21* | 0.485 | G | A | 0.489 | 1.42(1.12-1.79) | 3.4E-03 |  | 0.476 | 1.18(1.06-1.30) | 2.7E-03 |  | 0.478 | 1.21(1.10-1.33) | 6.7E-05 | 1.1E-02 | 0.149 | 0.520 | ++ |
| 1 | rs13103766 | 4 | 123517233 | intergenic | *IL2,IL21* | 0.485 | A | G | 0.488 | 1.43(1.13-1.80) | 2.8E-03 |  | 0.476 | 1.18(1.06-1.30) | 2.6E-03 |  | 0.478 | 1.22(1.10-1.33) | 5.7E-05 | 1.3E-02 | 0.133 | 0.556 | ++ |
| 0 | rs975403 | 4 | 123521640 | intergenic | *IL2,IL21* | 0.485 | A | G | 0.498 | 1.40(1.11-1.76) | 4.7E-03 |  | 0.478 | 1.18(1.06-1.30) | 2.8E-03 |  | 0.482 | 1.21(1.10-1.33) | 7.6E-05 | 7.4E-03 | 0.178 | 0.450 | ++ |
| 0 | rs11098667 | 4 | 123521682 | intergenic | *IL2,IL21* | 0.486 | G | A | 0.498 | 1.40(1.11-1.76) | 4.7E-03 |  | 0.478 | 1.18(1.06-1.30) | 2.8E-03 |  | 0.482 | 1.21(1.10-1.33) | 7.6E-05 | 7.4E-03 | 0.178 | 0.450 | ++ |
| 0 | rs6820964 | 4 | 123521723 | intergenic | *IL2,IL21* | 0.486 | A | G | 0.498 | 1.40(1.11-1.76) | 4.7E-03 |  | 0.478 | 1.18(1.06-1.30) | 2.8E-03 |  | 0.482 | 1.21(1.10-1.33) | 7.6E-05 | 7.4E-03 | 0.178 | 0.450 | ++ |
| 0 | rs6820791 | 4 | 123521783 | intergenic | *IL2,IL21* | 0.486 | T | C | 0.498 | 1.40(1.11-1.76) | 4.7E-03 |  | 0.478 | 1.18(1.06-1.30) | 2.8E-03 |  | 0.482 | 1.21(1.10-1.33) | 7.6E-05 | 7.4E-03 | 0.178 | 0.450 | ++ |
| 1 | rs28587458 | 4 | 162424701 | intronic | *FSTL5* | 0.738 | A | T | 0.722 | 1.47(1.14-1.90) | 3.7E-03 |  | 0.710 | 1.15(1.03-1.29) | 4.1E-03 |  | 0.712 | 1.20(1.08-1.33) | 5.9E-04 | 4.4E-02 | 0.094 | 0.643 | ++ |
| 0 | rs12517168 | 5 | 153099017 | intronic | *GRIA1* | 0.932 | A | G | 0.925 | 1.89(1.22-2.93) | 4.4E-03 |  | 0.930 | 1.32(1.08-1.61) | 6.9E-04 |  | 0.929 | 1.41(1.17-1.69) | 2.6E-04 | 1.9E-02 | 0.141 | 0.539 | ++ |
| 0 | rs11743471 | 5 | 153099883 | intronic | *GRIA1* | 0.933 | T | C | 0.925 | 1.97(1.27-3.06) | 2.6E-03 |  | 0.930 | 1.34(1.10-1.64) | 3.7E-04 |  | 0.929 | 1.44(1.20-1.73) | 1.1E-04 | 1.8E-02 | 0.120 | 0.587 | ++ |
| 0 | rs11747810 | 5 | 153100273 | intronic | *GRIA1* | 0.934 | C | T | 0.925 | 1.98(1.28-3.07) | 2.5E-03 |  | 0.931 | 1.33(1.09-1.63) | 5.7E-04 |  | 0.930 | 1.43(1.19-1.72) | 1.5E-04 | 2.3E-02 | 0.108 | 0.613 | ++ |
| 0 | rs6881133 | 5 | 153100545 | intronic | *GRIA1* | 0.934 | C | T | 0.925 | 2.01(1.30-3.11) | 1.9E-03 |  | 0.931 | 1.32(1.08-1.62) | 8.6E-04 |  | 0.930 | 1.42(1.18-1.71) | 1.8E-04 | 3.1E-02 | 0.088 | 0.657 | ++ |
| 0 | rs6859463 | 5 | 153100561 | intronic | *GRIA1* | 0.934 | G | A | 0.925 | 2.01(1.30-3.11) | 1.9E-03 |  | 0.931 | 1.33(1.09-1.63) | 5.7E-04 |  | 0.929 | 1.43(1.19-1.72) | 1.3E-04 | 2.6E-02 | 0.095 | 0.642 | ++ |
| 0 | rs6881792 | 5 | 153100871 | intronic | *GRIA1* | 0.933 | C | T | 0.925 | 2.01(1.30-3.11) | 1.9E-03 |  | 0.930 | 1.33(1.09-1.63) | 5.2E-04 |  | 0.929 | 1.44(1.19-1.72) | 1.2E-04 | 2.5E-02 | 0.096 | 0.640 | ++ |
| 0 | rs10067262 | 5 | 153101563 | intronic | *GRIA1* | 0.933 | T | C | 0.926 | 1.94(1.25-3.01) | 3.4E-03 |  | 0.930 | 1.34(1.10-1.64) | 3.7E-04 |  | 0.929 | 1.43(1.19-1.72) | 1.3E-04 | 1.6E-02 | 0.135 | 0.551 | ++ |
| 0 | rs6866103 | 5 | 153101915 | intronic | *GRIA1* | 0.934 | G | C | 0.925 | 1.98(1.28-3.07) | 2.5E-03 |  | 0.931 | 1.34(1.09-1.64) | 5.1E-04 |  | 0.930 | 1.43(1.19-1.72) | 1.3E-04 | 2.1E-02 | 0.111 | 0.605 | ++ |
| 0 | rs6891987 | 5 | 153102249 | intronic | *GRIA1* | 0.934 | C | T | 0.925 | 1.98(1.28-3.07) | 2.5E-03 |  | 0.931 | 1.34(1.09-1.64) | 5.1E-04 |  | 0.930 | 1.43(1.19-1.72) | 1.3E-04 | 2.1E-02 | 0.111 | 0.605 | ++ |
| 0 | rs7712550 | 5 | 153102951 | intronic | *GRIA1* | 0.908 | A | G | 0.916 | 1.86(1.21-2.85) | 4.7E-03 |  | 0.910 | 1.23(1.03-1.47) | 2.4E-03 |  | 0.911 | 1.30(1.11-1.54) | 1.3E-03 | 6.9E-02 | 0.079 | 0.676 | ++ |
| 0 | rs7712652 | 5 | 153102963 | intronic | *GRIA1* | 0.907 | A | G | 0.916 | 1.86(1.21-2.85) | 4.7E-03 |  | 0.900 | 1.22(1.03-1.46) | 3.0E-03 |  | 0.903 | 1.30(1.10-1.53) | 1.5E-03 | 7.4E-02 | 0.076 | 0.683 | ++ |
| 0 | rs7712284 | 5 | 153102979 | intronic | *GRIA1* | 0.906 | G | A | 0.915 | 1.86(1.22-2.85) | 4.6E-03 |  | 0.900 | 1.23(1.04-1.47) | 2.0E-03 |  | 0.903 | 1.31(1.11-1.54) | 1.1E-03 | 6.6E-02 | 0.080 | 0.674 | ++ |
| 0 | rs10071300 | 5 | 153103121 | intronic | *GRIA1* | 0.933 | C | G | 0.924 | 1.96(1.27-3.03) | 2.6E-03 |  | 0.930 | 1.32(1.08-1.62) | 7.3E-04 |  | 0.929 | 1.42(1.18-1.70) | 1.8E-04 | 2.4E-02 | 0.108 | 0.613 | ++ |
| 0 | rs10071192 | 5 | 153103156 | intronic | *GRIA1* | 0.933 | G | C | 0.925 | 1.93(1.25-2.98) | 3.3E-03 |  | 0.930 | 1.32(1.08-1.62) | 7.3E-04 |  | 0.929 | 1.41(1.18-1.70) | 2.1E-04 | 2.2E-02 | 0.123 | 0.581 | ++ |
| 1 | rs13167926 | 5 | 153103185 | intronic | *GRIA1* | 0.931 | A | G | 0.924 | 1.93(1.25-2.98) | 3.4E-03 |  | 0.930 | 1.34(1.10-1.64) | 3.5E-04 |  | 0.929 | 1.43(1.19-1.71) | 1.2E-04 | 1.5E-02 | 0.138 | 0.545 | ++ |
| 0 | rs6876587 | 5 | 153103261 | intronic | *GRIA1* | 0.924 | A | G | 0.921 | 1.86(1.21-2.86) | 4.8E-03 |  | 0.921 | 1.26(1.04-1.53) | 1.7E-03 |  | 0.921 | 1.35(1.13-1.60) | 8.1E-04 | 4.3E-02 | 0.106 | 0.618 | ++ |
| 0 | rs6876631 | 5 | 153103424 | intronic | *GRIA1* | 0.930 | A | C | 0.923 | 2.00(1.30-3.08) | 1.9E-03 |  | 0.928 | 1.30(1.07-1.59) | 1.2E-03 |  | 0.927 | 1.40(1.17-1.68) | 2.4E-04 | 3.8E-02 | 0.078 | 0.677 | ++ |
| 0 | rs7717329 | 5 | 153103954 | intronic | *GRIA1* | 0.933 | G | A | 0.924 | 1.96(1.27-3.03) | 2.6E-03 |  | 0.930 | 1.32(1.08-1.61) | 8.4E-04 |  | 0.929 | 1.42(1.18-1.70) | 2.0E-04 | 2.6E-02 | 0.106 | 0.618 | ++ |
| 0 | rs145569281 | 5 | 153104565 | intronic | *GRIA1* | 0.932 | A | G | 0.924 | 1.92(1.25-2.97) | 3.5E-03 |  | 0.930 | 1.33(1.08-1.62) | 6.2E-04 |  | 0.929 | 1.42(1.18-1.70) | 1.9E-04 | 2.0E-02 | 0.128 | 0.568 | ++ |
| 0 | rs199613155 | 5 | 153104638 | intronic | *GRIA1* | 0.932 | T | C | 0.924 | 1.92(1.25-2.97) | 3.5E-03 |  | 0.930 | 1.33(1.08-1.62) | 6.2E-04 |  | 0.929 | 1.42(1.18-1.70) | 1.9E-04 | 2.0E-02 | 0.128 | 0.568 | ++ |
| 0 | rs200925174 | 5 | 153104664 | intronic | *GRIA1* | 0.928 | G | C | 0.921 | 1.97(1.28-3.03) | 2.2E-03 |  | 0.923 | 1.30(1.07-1.58) | 8.9E-04 |  | 0.922 | 1.40(1.17-1.67) | 2.0E-04 | 3.5E-02 | 0.085 | 0.662 | ++ |
| 0 | rs142254982 | 5 | 153105708 | intronic | *GRIA1* | 0.933 | G | C | 0.924 | 1.96(1.27-3.03) | 2.6E-03 |  | 0.930 | 1.32(1.08-1.61) | 8.4E-04 |  | 0.929 | 1.42(1.18-1.70) | 2.0E-04 | 2.6E-02 | 0.105 | 0.619 | ++ |
| 0 | rs202053781 | 5 | 153105916 | intronic | *GRIA1* | 0.927 | C | G | 0.922 | 1.91(1.24-2.94) | 3.7E-03 |  | 0.926 | 1.27(1.04-1.54) | 2.1E-03 |  | 0.925 | 1.36(1.14-1.63) | 7.4E-04 | 4.6E-02 | 0.093 | 0.646 | ++ |
| 0 | rs200570844 | 5 | 153105920 | intronic | *GRIA1* | 0.932 | T | A | 0.923 | 1.92(1.25-2.96) | 3.3E-03 |  | 0.930 | 1.32(1.08-1.61) | 8.5E-04 |  | 0.929 | 1.41(1.18-1.69) | 2.1E-04 | 2.1E-02 | 0.123 | 0.579 | ++ |
| 0 | rs201037161 | 5 | 153106013 | intronic | *GRIA1* | 0.928 | G | C | 0.923 | 1.98(1.28-3.06) | 2.3E-03 |  | 0.925 | 1.31(1.08-1.59) | 9.0E-04 |  | 0.925 | 1.40(1.18-1.68) | 1.9E-04 | 3.2E-02 | 0.091 | 0.649 | ++ |
| **Lead** | **SNP** | **CHR** | **Position** | **Function** | **Gene** | **1KG_EAS** | **EA** | **NEA** | **GeneID-I (N=685)** | | |  | **NHAPC (N=2894)** | | |  | **Meta-analysis of GeneID-I and NHAPC (N=3579)** | | | | | | |
|  |  |  |  |  |  |  |  |  | **EAF** | **OR (95%CI)** | ***P*** |  | **EAF** | **OR (95%CI)** | ***P*** |  | **EAF** | **OR (95%CI)** | ***P*fixed** | ***P*random** | **Q** | **I2** | **Direction** |
| 0 | rs190301182 | 5 | 153106197 | intronic | *GRIA1* | 0.927 | G | A | 0.924 | 1.91(1.24-2.95) | 3.9E-03 |  | 0.924 | 1.30(1.07-1.58) | 9.8E-04 |  | 0.924 | 1.39(1.16-1.66) | 3.0E-04 | 2.8E-02 | 0.116 | 0.594 | ++ |
| 0 | rs78937211 | 5 | 153106457 | intronic | *GRIA1* | 0.932 | A | T | 0.924 | 1.95(1.26-3.01) | 2.8E-03 |  | 0.930 | 1.32(1.08-1.61) | 9.5E-04 |  | 0.928 | 1.41(1.18-1.69) | 2.3E-04 | 2.7E-02 | 0.107 | 0.615 | ++ |
| 0 | rs144182386 | 5 | 153106645 | intronic | *GRIA1* | 0.933 | C | T | 0.924 | 2.00(1.29-3.10) | 2.1E-03 |  | 0.929 | 1.32(1.08-1.61) | 8.1E-04 |  | 0.928 | 1.42(1.18-1.70) | 1.7E-04 | 3.0E-02 | 0.091 | 0.650 | ++ |
| 0 | rs9686257 | 5 | 153107260 | intronic | *GRIA1* | 0.925 | G | A | 0.919 | 1.88(1.24-2.85) | 3.4E-03 |  | 0.923 | 1.26(1.04-1.53) | 2.0E-03 |  | 0.922 | 1.35(1.13-1.61) | 7.9E-04 | 4.8E-02 | 0.089 | 0.653 | ++ |
| 0 | rs9687431 | 5 | 153107349 | intronic | *GRIA1* | 0.931 | A | G | 0.924 | 1.92(1.25-2.97) | 3.5E-03 |  | 0.928 | 1.31(1.07-1.59) | 1.1E-03 |  | 0.927 | 1.40(1.16-1.67) | 3.0E-04 | 2.8E-02 | 0.112 | 0.603 | ++ |
| 0 | rs9687441 | 5 | 153107480 | intronic | *GRIA1* | 0.929 | A | G | 0.922 | 1.92(1.25-2.96) | 3.2E-03 |  | 0.926 | 1.29(1.06-1.57) | 1.4E-03 |  | 0.925 | 1.38(1.16-1.66) | 3.9E-04 | 3.5E-02 | 0.100 | 0.631 | ++ |
| 0 | rs13166799 | 5 | 153108631 | intronic | *GRIA1* | 0.930 | T | C | 0.921 | 1.93(1.25-2.98) | 3.2E-03 |  | 0.924 | 1.30(1.07-1.58) | 6.7E-04 |  | 0.923 | 1.39(1.17-1.66) | 2.6E-04 | 3.1E-02 | 0.104 | 0.621 | ++ |
| 0 | rs13166807 | 5 | 153108685 | intronic | *GRIA1* | 0.932 | G | A | 0.923 | 1.95(1.26-3.01) | 2.8E-03 |  | 0.929 | 1.32(1.08-1.61) | 7.8E-04 |  | 0.928 | 1.41(1.18-1.70) | 2.0E-04 | 2.5E-02 | 0.110 | 0.609 | ++ |
| 0 | rs7736016 | 5 | 153108886 | intronic | *GRIA1* | 0.933 | C | T | 0.924 | 1.96(1.27-3.03) | 2.6E-03 |  | 0.930 | 1.32(1.08-1.61) | 8.4E-04 |  | 0.929 | 1.42(1.18-1.70) | 2.0E-04 | 2.6E-02 | 0.105 | 0.619 | ++ |
| 0 | rs7716290 | 5 | 153108974 | intronic | *GRIA1* | 0.933 | A | G | 0.925 | 1.93(1.25-2.98) | 3.3E-03 |  | 0.930 | 1.31(1.07-1.61) | 9.4E-04 |  | 0.929 | 1.41(1.17-1.69) | 2.8E-04 | 2.6E-02 | 0.115 | 0.597 | ++ |
| 0 | rs7720354 | 5 | 153109185 | intronic | *GRIA1* | 0.933 | T | G | 0.923 | 1.92(1.25-2.95) | 3.3E-03 |  | 0.930 | 1.32(1.08-1.61) | 8.4E-04 |  | 0.929 | 1.41(1.18-1.69) | 2.2E-04 | 2.1E-02 | 0.124 | 0.577 | ++ |
| 0 | rs10477084 | 5 | 153109857 | intronic | *GRIA1* | 0.933 | A | G | 0.920 | 1.84(1.21-2.81) | 4.9E-03 |  | 0.930 | 1.32(1.08-1.61) | 8.7E-04 |  | 0.928 | 1.40(1.17-1.68) | 2.7E-04 | 1.4E-02 | 0.161 | 0.490 | ++ |
| 0 | rs10477085 | 5 | 153109957 | intronic | *GRIA1* | 0.933 | G | A | 0.923 | 1.92(1.25-2.95) | 3.3E-03 |  | 0.930 | 1.32(1.08-1.61) | 8.4E-04 |  | 0.929 | 1.41(1.18-1.69) | 2.2E-04 | 2.1E-02 | 0.124 | 0.577 | ++ |
| 0 | rs6580030 | 5 | 153110178 | intronic | *GRIA1* | 0.933 | G | A | 0.923 | 1.92(1.25-2.95) | 3.3E-03 |  | 0.930 | 1.32(1.08-1.61) | 8.4E-04 |  | 0.929 | 1.41(1.18-1.69) | 2.2E-04 | 2.1E-02 | 0.124 | 0.577 | ++ |
| 0 | rs6580031 | 5 | 153110240 | intronic | *GRIA1* | 0.933 | A | G | 0.923 | 1.92(1.25-2.95) | 3.3E-03 |  | 0.930 | 1.32(1.08-1.61) | 8.4E-04 |  | 0.929 | 1.41(1.18-1.69) | 2.2E-04 | 2.1E-02 | 0.124 | 0.577 | ++ |
| 1 | rs6580032 | 5 | 153110288 | intronic | *GRIA1* | 0.933 | G | A | 0.917 | 2.17(1.42-3.32) | 3.5E-04 |  | 0.930 | 1.32(1.08-1.61) | 8.4E-04 |  | 0.927 | 1.45(1.21-1.74) | 6.9E-05 | 4.7E-02 | 0.037 | 0.771 | ++ |
| 0 | rs6580033 | 5 | 153110458 | intronic | *GRIA1* | 0.933 | G | A | 0.925 | 1.93(1.25-2.98) | 3.3E-03 |  | 0.930 | 1.31(1.07-1.61) | 9.4E-04 |  | 0.929 | 1.41(1.17-1.69) | 2.8E-04 | 2.6E-02 | 0.115 | 0.597 | ++ |
| 0 | rs6886614 | 5 | 153110621 | intronic | *GRIA1* | 0.933 | G | C | 0.925 | 1.93(1.25-2.98) | 3.3E-03 |  | 0.930 | 1.32(1.08-1.61) | 8.4E-04 |  | 0.929 | 1.41(1.18-1.70) | 2.3E-04 | 2.3E-02 | 0.120 | 0.586 | ++ |
| 0 | rs6580034 | 5 | 153110646 | intronic | *GRIA1* | 0.933 | G | C | 0.925 | 1.93(1.25-2.98) | 3.3E-03 |  | 0.930 | 1.32(1.08-1.61) | 8.4E-04 |  | 0.929 | 1.41(1.18-1.70) | 2.3E-04 | 2.3E-02 | 0.120 | 0.586 | ++ |
| 0 | rs7731036 | 5 | 153111401 | intronic | *GRIA1* | 0.933 | G | A | 0.925 | 1.92(1.25-2.97) | 3.5E-03 |  | 0.930 | 1.31(1.07-1.61) | 9.4E-04 |  | 0.929 | 1.40(1.17-1.69) | 2.8E-04 | 2.5E-02 | 0.118 | 0.591 | ++ |
| 0 | rs7731539 | 5 | 153111661 | intronic | *GRIA1* | 0.933 | G | C | 0.923 | 1.89(1.22-2.93) | 4.4E-03 |  | 0.920 | 1.30(1.07-1.59) | 6.5E-04 |  | 0.921 | 1.39(1.16-1.66) | 3.1E-04 | 2.5E-02 | 0.127 | 0.570 | ++ |
| 0 | rs7731561 | 5 | 153111736 | intronic | *GRIA1* | 0.932 | G | A | 0.925 | 1.93(1.25-2.98) | 3.3E-03 |  | 0.930 | 1.32(1.08-1.61) | 8.4E-04 |  | 0.929 | 1.41(1.18-1.70) | 2.3E-04 | 2.3E-02 | 0.120 | 0.586 | ++ |
| 0 | rs4644006 | 5 | 153112500 | intronic | *GRIA1* | 0.932 | T | C | 0.925 | 1.97(1.27-3.05) | 2.7E-03 |  | 0.930 | 1.32(1.08-1.62) | 7.6E-04 |  | 0.929 | 1.42(1.18-1.70) | 2.1E-04 | 2.7E-02 | 0.106 | 0.618 | ++ |
| 0 | rs6869815 | 5 | 153114843 | intronic | *GRIA1* | 0.932 | A | G | 0.925 | 1.93(1.25-2.98) | 3.4E-03 |  | 0.930 | 1.32(1.08-1.61) | 7.7E-04 |  | 0.929 | 1.41(1.18-1.70) | 2.3E-04 | 2.3E-02 | 0.121 | 0.583 | ++ |
| 0 | rs6580036 | 5 | 153115963 | intronic | *GRIA1* | 0.931 | C | A | 0.925 | 1.93(1.25-2.98) | 3.4E-03 |  | 0.930 | 1.31(1.07-1.60) | 8.3E-04 |  | 0.929 | 1.40(1.17-1.68) | 2.9E-04 | 2.7E-02 | 0.115 | 0.598 | ++ |
| 0 | rs4377754 | 5 | 153116675 | intronic | *GRIA1* | 0.931 | C | T | 0.925 | 1.93(1.25-2.98) | 3.4E-03 |  | 0.930 | 1.31(1.07-1.60) | 8.3E-04 |  | 0.929 | 1.40(1.17-1.68) | 2.9E-04 | 2.7E-02 | 0.115 | 0.598 | ++ |
| 0 | rs6896622 | 5 | 153117507 | intronic | *GRIA1* | 0.931 | A | G | 0.925 | 1.93(1.25-2.98) | 3.4E-03 |  | 0.930 | 1.31(1.07-1.60) | 8.3E-04 |  | 0.929 | 1.40(1.17-1.68) | 2.9E-04 | 2.7E-02 | 0.115 | 0.598 | ++ |
| 0 | rs6897016 | 5 | 153117936 | intronic | *GRIA1* | 0.931 | T | C | 0.925 | 1.93(1.25-2.99) | 3.2E-03 |  | 0.929 | 1.32(1.08-1.61) | 7.4E-04 |  | 0.928 | 1.41(1.17-1.69) | 2.3E-04 | 2.4E-02 | 0.117 | 0.593 | ++ |
| 0 | rs6861421 | 5 | 153118443 | intronic | *GRIA1* | 0.929 | T | C | 0.924 | 1.88(1.22-2.90) | 4.5E-03 |  | 0.930 | 1.30(1.07-1.59) | 9.6E-04 |  | 0.929 | 1.39(1.16-1.66) | 3.7E-04 | 2.5E-02 | 0.130 | 0.563 | ++ |
| 0 | rs4273649 | 5 | 153120048 | intronic | *GRIA1* | . | G | A | 0.925 | 1.93(1.25-2.98) | 3.4E-03 |  | 0.930 | 1.31(1.07-1.60) | 9.1E-04 |  | 0.929 | 1.40(1.17-1.68) | 3.0E-04 | 2.7E-02 | 0.114 | 0.599 | ++ |
| 0 | rs6421143 | 5 | 153120718 | intronic | *GRIA1* | 0.931 | G | A | 0.924 | 1.88(1.22-2.90) | 4.5E-03 |  | 0.930 | 1.33(1.09-1.62) | 4.6E-04 |  | 0.929 | 1.41(1.18-1.69) | 1.9E-04 | 1.4E-02 | 0.154 | 0.509 | ++ |
| 0 | rs6870761 | 5 | 153123344 | intronic | *GRIA1* | 0.931 | G | T | 0.925 | 1.93(1.25-2.98) | 3.4E-03 |  | 0.930 | 1.30(1.06-1.59) | 1.1E-03 |  | 0.929 | 1.40(1.16-1.68) | 3.6E-04 | 3.0E-02 | 0.109 | 0.610 | ++ |
| 0 | rs6889794 | 5 | 153123455 | intronic | *GRIA1* | 0.931 | G | A | 0.925 | 1.93(1.25-2.98) | 3.4E-03 |  | 0.930 | 1.30(1.06-1.59) | 1.1E-03 |  | 0.929 | 1.40(1.16-1.68) | 3.6E-04 | 3.0E-02 | 0.109 | 0.610 | ++ |
| 0 | rs6890276 | 5 | 153123729 | intronic | *GRIA1* | 0.931 | G | A | 0.925 | 1.93(1.25-2.98) | 3.4E-03 |  | 0.930 | 1.30(1.06-1.59) | 1.1E-03 |  | 0.929 | 1.40(1.16-1.68) | 3.6E-04 | 3.0E-02 | 0.109 | 0.610 | ++ |
| 0 | rs4958675 | 5 | 153124488 | intronic | *GRIA1* | 0.931 | C | T | 0.925 | 1.93(1.25-2.98) | 3.4E-03 |  | 0.930 | 1.30(1.06-1.59) | 1.1E-03 |  | 0.929 | 1.40(1.16-1.68) | 3.6E-04 | 3.0E-02 | 0.109 | 0.610 | ++ |
| 0 | rs10063691 | 5 | 153124798 | intronic | *GRIA1* | 0.931 | G | C | 0.925 | 1.93(1.25-2.98) | 3.4E-03 |  | 0.930 | 1.30(1.06-1.59) | 1.1E-03 |  | 0.929 | 1.40(1.16-1.68) | 3.6E-04 | 3.0E-02 | 0.109 | 0.610 | ++ |
| 0 | rs10071736 | 5 | 153124963 | intronic | *GRIA1* | 0.931 | C | T | 0.925 | 1.93(1.25-2.98) | 3.4E-03 |  | 0.930 | 1.30(1.06-1.59) | 1.1E-03 |  | 0.929 | 1.40(1.16-1.68) | 3.6E-04 | 3.0E-02 | 0.109 | 0.610 | ++ |
| 0 | rs4645377 | 5 | 153126156 | intronic | *GRIA1* | 0.928 | A | C | 0.923 | 1.86(1.22-2.86) | 4.7E-03 |  | 0.930 | 1.30(1.07-1.59) | 9.9E-04 |  | 0.929 | 1.39(1.16-1.66) | 3.8E-04 | 2.3E-02 | 0.136 | 0.551 | ++ |
| 0 | rs7707437 | 5 | 153126223 | intronic | *GRIA1* | 0.928 | A | G | 0.923 | 1.86(1.22-2.86) | 4.7E-03 |  | 0.930 | 1.30(1.07-1.59) | 9.9E-04 |  | 0.929 | 1.39(1.16-1.66) | 3.8E-04 | 2.3E-02 | 0.136 | 0.551 | ++ |
| 0 | rs11167639 | 5 | 153126615 | intronic | *GRIA1* | 0.928 | A | G | 0.923 | 1.86(1.22-2.86) | 4.7E-03 |  | 0.930 | 1.30(1.07-1.59) | 9.9E-04 |  | 0.929 | 1.39(1.16-1.66) | 3.8E-04 | 2.3E-02 | 0.136 | 0.551 | ++ |
| **Lead** | **SNP** | **CHR** | **Position** | **Function** | **Gene** | **1KG_EAS** | **EA** | **NEA** | **GeneID-I (N=685)** | | |  | **NHAPC (N=2894)** | | |  | **Meta-analysis of GeneID-I and NHAPC (N=3579)** | | | | | | |
|  |  |  |  |  |  |  |  |  | **EAF** | **OR (95%CI)** | ***P*** |  | **EAF** | **OR (95%CI)** | ***P*** |  | **EAF** | **OR (95%CI)** | ***P*fixed** | ***P*random** | **Q** | **I2** | **Direction** |
| 0 | rs4128572 | 5 | 153128715 | intronic | *GRIA1* | 0.931 | G | A | 0.925 | 1.93(1.25-2.98) | 3.4E-03 |  | 0.930 | 1.30(1.06-1.59) | 1.1E-03 |  | 0.929 | 1.40(1.16-1.68) | 3.6E-04 | 3.0E-02 | 0.109 | 0.610 | ++ |
| 0 | rs6580037 | 5 | 153132632 | intronic | *GRIA1* | 0.928 | G | C | 0.923 | 1.86(1.22-2.86) | 4.7E-03 |  | 0.930 | 1.31(1.07-1.60) | 8.8E-04 |  | 0.929 | 1.39(1.16-1.67) | 3.2E-04 | 2.0E-02 | 0.141 | 0.539 | ++ |
| 0 | rs6580038 | 5 | 153132841 | intronic | *GRIA1* | 0.931 | T | C | 0.925 | 1.93(1.25-2.98) | 3.4E-03 |  | 0.930 | 1.30(1.06-1.59) | 1.1E-03 |  | 0.929 | 1.40(1.16-1.68) | 3.6E-04 | 3.0E-02 | 0.109 | 0.610 | ++ |
| 0 | rs11748890 | 5 | 153135406 | intronic | *GRIA1* | 0.931 | A | C | 0.925 | 1.93(1.25-2.98) | 3.4E-03 |  | 0.930 | 1.30(1.06-1.59) | 1.1E-03 |  | 0.929 | 1.40(1.16-1.68) | 3.6E-04 | 3.0E-02 | 0.109 | 0.610 | ++ |
| 0 | rs6893441 | 5 | 153136066 | intronic | *GRIA1* | 0.931 | A | G | 0.925 | 1.93(1.25-2.98) | 3.4E-03 |  | 0.930 | 1.30(1.06-1.59) | 1.1E-03 |  | 0.929 | 1.40(1.16-1.68) | 3.6E-04 | 3.0E-02 | 0.109 | 0.610 | ++ |
| 0 | rs7445290 | 5 | 153136702 | intronic | *GRIA1* | 0.931 | G | T | 0.925 | 1.93(1.25-2.98) | 3.4E-03 |  | 0.930 | 1.30(1.06-1.59) | 1.1E-03 |  | 0.929 | 1.40(1.16-1.68) | 3.6E-04 | 3.0E-02 | 0.109 | 0.610 | ++ |
| 0 | rs7445323 | 5 | 153136785 | intronic | *GRIA1* | 0.931 | G | T | 0.925 | 1.93(1.25-2.98) | 3.4E-03 |  | 0.930 | 1.30(1.06-1.59) | 1.1E-03 |  | 0.929 | 1.40(1.16-1.68) | 3.6E-04 | 3.0E-02 | 0.109 | 0.610 | ++ |
| 0 | rs7709205 | 5 | 153138709 | intronic | *GRIA1* | 0.931 | C | T | 0.925 | 1.93(1.25-2.98) | 3.4E-03 |  | 0.930 | 1.30(1.06-1.59) | 1.1E-03 |  | 0.929 | 1.40(1.16-1.68) | 3.6E-04 | 3.0E-02 | 0.109 | 0.610 | ++ |
| 0 | rs7731986 | 5 | 153139614 | intronic | *GRIA1* | 0.931 | G | A | 0.925 | 1.93(1.25-2.98) | 3.4E-03 |  | 0.930 | 1.30(1.06-1.59) | 1.1E-03 |  | 0.929 | 1.40(1.16-1.68) | 3.6E-04 | 3.0E-02 | 0.109 | 0.610 | ++ |
| 0 | rs6580039 | 5 | 153142122 | intronic | *GRIA1* | 0.931 | T | C | 0.925 | 1.93(1.25-2.98) | 3.4E-03 |  | 0.930 | 1.30(1.06-1.59) | 1.1E-03 |  | 0.929 | 1.40(1.16-1.68) | 3.6E-04 | 3.0E-02 | 0.109 | 0.610 | ++ |
| 0 | rs7711124 | 5 | 153142894 | intronic | *GRIA1* | 0.928 | C | G | 0.923 | 1.87(1.22-2.87) | 4.6E-03 |  | 0.928 | 1.30(1.07-1.59) | 9.9E-04 |  | 0.927 | 1.39(1.16-1.66) | 3.7E-04 | 2.3E-02 | 0.134 | 0.554 | ++ |
| 0 | rs3813470 | 5 | 153144216 | intronic | *GRIA1* | 0.931 | G | A | 0.925 | 1.93(1.25-2.98) | 3.4E-03 |  | 0.930 | 1.30(1.06-1.59) | 1.1E-03 |  | 0.929 | 1.40(1.16-1.68) | 3.6E-04 | 3.0E-02 | 0.109 | 0.610 | ++ |
| 0 | rs12717883 | 5 | 153144835 | intronic | *GRIA1* | 0.931 | G | A | 0.925 | 1.93(1.25-2.98) | 3.4E-03 |  | 0.930 | 1.30(1.06-1.59) | 1.1E-03 |  | 0.929 | 1.40(1.16-1.68) | 3.6E-04 | 3.0E-02 | 0.109 | 0.610 | ++ |
| 0 | rs4254956 | 5 | 153145184 | intronic | *GRIA1* | 0.931 | C | G | 0.925 | 1.93(1.25-2.98) | 3.4E-03 |  | 0.930 | 1.30(1.06-1.59) | 1.1E-03 |  | 0.929 | 1.40(1.16-1.68) | 3.6E-04 | 3.0E-02 | 0.109 | 0.610 | ++ |
| 0 | rs11749611 | 5 | 153146418 | intronic | *GRIA1* | 0.931 | A | G | 0.925 | 1.93(1.25-2.98) | 3.4E-03 |  | 0.930 | 1.30(1.06-1.59) | 1.1E-03 |  | 0.929 | 1.40(1.16-1.68) | 3.6E-04 | 3.0E-02 | 0.109 | 0.610 | ++ |
| 0 | rs4576205 | 5 | 153147848 | intronic | *GRIA1* | 0.931 | T | C | 0.925 | 1.93(1.25-2.98) | 3.4E-03 |  | 0.930 | 1.30(1.06-1.59) | 1.1E-03 |  | 0.929 | 1.40(1.16-1.68) | 3.6E-04 | 3.0E-02 | 0.109 | 0.610 | ++ |
| 0 | rs2964018 | 5 | 153150540 | intronic | *GRIA1* | 0.931 | G | A | 0.925 | 1.93(1.25-2.98) | 3.4E-03 |  | 0.930 | 1.30(1.06-1.59) | 1.1E-03 |  | 0.929 | 1.40(1.16-1.68) | 3.6E-04 | 3.0E-02 | 0.109 | 0.610 | ++ |
| 0 | rs2926847 | 5 | 153151792 | intronic | *GRIA1* | 0.931 | C | T | 0.925 | 1.93(1.25-2.98) | 3.4E-03 |  | 0.930 | 1.30(1.06-1.59) | 1.1E-03 |  | 0.929 | 1.40(1.16-1.68) | 3.6E-04 | 3.0E-02 | 0.109 | 0.610 | ++ |
| 0 | rs57001955 | 5 | 153153642 | intronic | *GRIA1* | 0.928 | C | A | 0.925 | 1.90(1.23-2.95) | 4.1E-03 |  | 0.930 | 1.31(1.07-1.60) | 9.7E-04 |  | 0.929 | 1.40(1.16-1.67) | 3.3E-04 | 2.6E-02 | 0.124 | 0.577 | ++ |
| 0 | rs2926849 | 5 | 153156670 | intronic | *GRIA1* | 0.931 | C | T | 0.925 | 1.93(1.25-2.98) | 3.4E-03 |  | 0.930 | 1.30(1.06-1.60) | 1.0E-03 |  | 0.929 | 1.40(1.16-1.68) | 3.5E-04 | 3.0E-02 | 0.110 | 0.608 | ++ |
| 0 | rs2964016 | 5 | 153156719 | intronic | *GRIA1* | 0.931 | A | G | 0.925 | 1.93(1.25-2.98) | 3.4E-03 |  | 0.930 | 1.30(1.07-1.60) | 1.0E-03 |  | 0.929 | 1.40(1.16-1.68) | 3.4E-04 | 2.9E-02 | 0.111 | 0.607 | ++ |
| 0 | rs2964024 | 5 | 153158201 | intronic | *GRIA1* | 0.933 | C | T | 0.925 | 1.92(1.24-2.97) | 3.6E-03 |  | 0.930 | 1.30(1.06-1.60) | 9.9E-04 |  | 0.929 | 1.40(1.16-1.68) | 3.6E-04 | 2.9E-02 | 0.115 | 0.598 | ++ |
| 0 | rs2926853 | 5 | 153159284 | intronic | *GRIA1* | 0.933 | G | A | 0.925 | 1.92(1.24-2.97) | 3.6E-03 |  | 0.930 | 1.31(1.07-1.60) | 8.7E-04 |  | 0.929 | 1.40(1.17-1.68) | 3.2E-04 | 2.6E-02 | 0.118 | 0.591 | ++ |
| 0 | rs1461231 | 5 | 153161149 | intronic | *GRIA1* | 0.933 | C | G | 0.925 | 1.92(1.24-2.97) | 3.6E-03 |  | 0.930 | 1.31(1.07-1.60) | 9.0E-04 |  | 0.929 | 1.40(1.16-1.68) | 3.3E-04 | 2.7E-02 | 0.117 | 0.592 | ++ |
| 0 | rs1461232 | 5 | 153162173 | intronic | *GRIA1* | 0.933 | G | C | 0.924 | 1.91(1.24-2.95) | 3.8E-03 |  | 0.930 | 1.30(1.06-1.59) | 9.7E-04 |  | 0.929 | 1.40(1.16-1.68) | 3.7E-04 | 2.8E-02 | 0.118 | 0.590 | ++ |
| 0 | rs2964026 | 5 | 153162394 | intronic | *GRIA1* | 0.933 | A | G | 0.925 | 1.92(1.24-2.97) | 3.6E-03 |  | 0.930 | 1.31(1.07-1.60) | 9.0E-04 |  | 0.929 | 1.40(1.16-1.68) | 3.3E-04 | 2.7E-02 | 0.117 | 0.592 | ++ |
| 0 | rs2926854 | 5 | 153163044 | intronic | *GRIA1* | 0.933 | A | T | 0.925 | 1.92(1.24-2.97) | 3.6E-03 |  | 0.930 | 1.31(1.07-1.60) | 9.0E-04 |  | 0.929 | 1.40(1.16-1.68) | 3.3E-04 | 2.7E-02 | 0.117 | 0.592 | ++ |
| 0 | rs1461233 | 5 | 153163653 | intronic | *GRIA1* | 0.933 | C | A | 0.925 | 1.92(1.24-2.97) | 3.6E-03 |  | 0.930 | 1.31(1.07-1.60) | 9.0E-04 |  | 0.929 | 1.40(1.16-1.68) | 3.3E-04 | 2.7E-02 | 0.117 | 0.592 | ++ |
| 0 | rs2926855 | 5 | 153164250 | intronic | *GRIA1* | 0.933 | A | C | 0.925 | 1.92(1.24-2.97) | 3.6E-03 |  | 0.930 | 1.31(1.07-1.60) | 9.0E-04 |  | 0.929 | 1.40(1.16-1.68) | 3.3E-04 | 2.7E-02 | 0.117 | 0.592 | ++ |
| 0 | rs2926856 | 5 | 153165949 | intronic | *GRIA1* | 0.933 | T | C | 0.925 | 1.92(1.24-2.97) | 3.6E-03 |  | 0.930 | 1.31(1.07-1.60) | 9.0E-04 |  | 0.929 | 1.40(1.16-1.68) | 3.3E-04 | 2.7E-02 | 0.117 | 0.592 | ++ |
| 0 | rs2926869 | 5 | 153166884 | intronic | *GRIA1* | 0.933 | G | A | 0.925 | 1.92(1.24-2.97) | 3.6E-03 |  | 0.930 | 1.31(1.07-1.60) | 8.7E-04 |  | 0.929 | 1.40(1.17-1.68) | 3.2E-04 | 2.6E-02 | 0.118 | 0.590 | ++ |
| 0 | rs2126165 | 5 | 153167595 | intronic | *GRIA1* | 0.933 | G | A | 0.925 | 1.92(1.24-2.97) | 3.6E-03 |  | 0.930 | 1.31(1.07-1.61) | 7.8E-04 |  | 0.929 | 1.40(1.17-1.69) | 2.8E-04 | 2.4E-02 | 0.121 | 0.583 | ++ |
| 0 | rs2963999 | 5 | 153176073 | intronic | *GRIA1* | 0.933 | T | G | 0.925 | 1.92(1.24-2.97) | 3.6E-03 |  | 0.930 | 1.33(1.09-1.63) | 4.9E-04 |  | 0.929 | 1.42(1.18-1.71) | 1.7E-04 | 1.7E-02 | 0.135 | 0.552 | ++ |
| 0 | rs2963998 | 5 | 153177053 | intronic | *GRIA1* | 0.933 | T | C | 0.925 | 1.92(1.24-2.97) | 3.6E-03 |  | 0.930 | 1.32(1.07-1.61) | 7.2E-04 |  | 0.929 | 1.41(1.17-1.69) | 2.6E-04 | 2.3E-02 | 0.124 | 0.578 | ++ |
| 0 | rs2964005 | 5 | 153214011 | intergenic | *GRIA1,FAM114A2* | 0.933 | C | G | 0.924 | 1.89(1.23-2.91) | 4.3E-03 |  | 0.929 | 1.29(1.05-1.57) | 1.6E-03 |  | 0.928 | 1.38(1.15-1.65) | 5.6E-04 | 3.3E-02 | 0.116 | 0.595 | ++ |
| 0 | rs2964004 | 5 | 153215342 | intergenic | *GRIA1,FAM114A2* | 0.933 | G | A | 0.924 | 1.89(1.23-2.91) | 4.3E-03 |  | 0.929 | 1.28(1.05-1.57) | 1.7E-03 |  | 0.928 | 1.38(1.15-1.65) | 6.1E-04 | 3.5E-02 | 0.113 | 0.601 | ++ |
| 0 | rs849295 | 6 | 141185275 | intergenic | *MIR4465,NMBR* | 0.815 | C | G | 0.171 | 1.53(1.15-2.03) | 3.3E-03 |  | 0.170 | 1.24(1.07-1.42) | 3.4E-03 |  | 0.170 | 1.29(1.14-1.46) | 7.2E-05 | 4.6E-03 | 0.187 | 0.426 | ++ |
| 0 | rs7773958 | 6 | 141186594 | intergenic | *MIR4465,NMBR* | 0.186 | A | T | 0.171 | 1.53(1.15-2.03) | 3.3E-03 |  | 0.170 | 1.24(1.07-1.42) | 3.4E-03 |  | 0.170 | 1.29(1.14-1.46) | 7.2E-05 | 4.6E-03 | 0.187 | 0.426 | ++ |
| 1 | rs78521628 | 6 | 141192312 | intergenic | *MIR4465,NMBR* | 0.186 | T | C | 0.171 | 1.54(1.16-2.04) | 2.9E-03 |  | 0.166 | 1.24(1.07-1.42) | 3.3E-03 |  | 0.167 | 1.29(1.14-1.47) | 6.6E-05 | 5.4E-03 | 0.175 | 0.456 | ++ |
| 0 | rs6910195 | 6 | 141192610 | intergenic | *MIR4465,NMBR* | 0.290 | G | C | 0.271 | 1.47(1.15-1.89) | 2.5E-03 |  | 0.267 | 1.17(1.04-1.31) | 4.6E-03 |  | 0.268 | 1.22(1.10-1.36) | 2.5E-04 | 2.9E-02 | 0.100 | 0.631 | ++ |
| **Lead** | **SNP** | **CHR** | **Position** | **Function** | **Gene** | **1KG_EAS** | **EA** | **NEA** | **GeneID-I (N=685)** | | |  | **NHAPC (N=2894)** | | |  | **Meta-analysis of GeneID-I and NHAPC (N=3579)** | | | | | | |
|  |  |  |  |  |  |  |  |  | **EAF** | **OR (95%CI)** | ***P*** |  | **EAF** | **OR (95%CI)** | ***P*** |  | **EAF** | **OR (95%CI)** | ***P*fixed** | ***P*random** | **Q** | **I2** | **Direction** |
| 0 | rs11756619 | 6 | 141199815 | intergenic | *MIR4465,NMBR* | 0.186 | G | A | 0.171 | 1.54(1.16-2.04) | 2.9E-03 |  | 0.165 | 1.24(1.07-1.42) | 3.6E-03 |  | 0.166 | 1.29(1.14-1.46) | 7.4E-05 | 6.1E-03 | 0.171 | 0.467 | ++ |
| 0 | rs12204980 | 6 | 141217085 | intergenic | *MIR4465,NMBR* | 0.186 | A | G | 0.169 | 1.54(1.16-2.05) | 3.5E-03 |  | 0.165 | 1.23(1.07-1.42) | 3.6E-03 |  | 0.166 | 1.29(1.13-1.46) | 9.1E-05 | 6.7E-03 | 0.174 | 0.458 | ++ |
| 1 | rs10847208 | 12 | 127229506 | ncRNA_intronic | *LINC00943,LINC00944* | 0.861 | C | T | 0.843 | 1.58(1.18-2.13) | 2.6E-03 |  | 0.839 | 1.30(1.13-1.50) | 2.7E-04 |  | 0.840 | 1.35(1.19-1.53) | 4.0E-06 | 3.2E-04 | 0.239 | 0.279 | ++ |
| 0 | rs60773474 | 13 | 112097963 | intergenic | *TEX29,LINC00354* | 0.143 | G | A | 0.891 | 2.33(1.59-3.43) | 1.9E-05 |  | 0.880 | 1.20(1.03-1.41) | 4.9E-03 |  | 0.882 | 1.32(1.14-1.53) | 1.8E-04 | 1.4E-01 | 0.002 | 0.898 | ++ |
| 1 | rs74129462 | 13 | 112106861 | intergenic | *TEX29,LINC00354* | 0.143 | G | T | 0.891 | 2.31(1.57-3.39) | 2.4E-05 |  | 0.880 | 1.21(1.03-1.42) | 4.3E-03 |  | 0.882 | 1.33(1.15-1.53) | 1.5E-04 | 1.3E-01 | 0.002 | 0.893 | ++ |
| 0 | rs57049971 | 13 | 112110168 | intergenic | *TEX29,LINC00354* | 0.144 | C | A | 0.891 | 2.31(1.57-3.39) | 2.4E-05 |  | 0.880 | 1.21(1.03-1.41) | 4.7E-03 |  | 0.882 | 1.32(1.14-1.53) | 1.6E-04 | 1.3E-01 | 0.002 | 0.893 | ++ |
| 1 | rs1287404 | 14 | 89983256 | intronic | *FOXN3* | 0.194 | C | T | 0.779 | 1.49(1.15-1.93) | 2.8E-03 |  | 0.774 | 1.18(1.04-1.33) | 4.7E-03 |  | 0.775 | 1.23(1.10-1.38) | 2.5E-04 | 2.5E-02 | 0.112 | 0.603 | ++ |
| 1 | rs58232781 | 15 | 73105732 | intergenic | *ADPGK-AS1,NEO1* | 0.514 | G | A | 0.537 | 1.40(1.12-1.75) | 3.4E-03 |  | 0.525 | 1.15(1.04-1.28) | 2.8E-03 |  | 0.527 | 1.19(1.08-1.31) | 2.6E-04 | 2.5E-02 | 0.117 | 0.592 | ++ |
| 0 | rs2868465 | 19 | 28457604 | intergenic | *LOC101927151,LOC100420587* | 0.151 | C | A | 0.823 | 1.64(1.22-2.20) | 1.1E-03 |  | 0.838 | 1.19(1.03-1.36) | 3.9E-03 |  | 0.835 | 1.26(1.11-1.43) | 3.6E-04 | 5.5E-02 | 0.052 | 0.734 | ++ |
| 0 | rs76096927 | 19 | 28458048 | intergenic | *LOC101927151,LOC100420587* | 0.151 | T | G | 0.821 | 1.61(1.20-2.17) | 1.6E-03 |  | 0.838 | 1.19(1.03-1.36) | 3.8E-03 |  | 0.835 | 1.26(1.11-1.42) | 4.2E-04 | 5.0E-02 | 0.064 | 0.708 | ++ |
| 0 | rs75070951 | 19 | 28462118 | intergenic | *LOC101927151,LOC100420587* | 0.151 | C | A | 0.823 | 1.65(1.23-2.21) | 9.1E-04 |  | 0.838 | 1.19(1.03-1.36) | 3.9E-03 |  | 0.835 | 1.26(1.11-1.43) | 3.3E-04 | 5.8E-02 | 0.046 | 0.748 | ++ |
| 1 | rs2126982 | 19 | 28475962 | intergenic | *LOC101927151,LOC100420587* | 0.151 | G | C | 0.826 | 1.61(1.20-2.16) | 1.8E-03 |  | 0.839 | 1.19(1.03-1.37) | 3.6E-03 |  | 0.836 | 1.26(1.11-1.42) | 4.4E-04 | 4.8E-02 | 0.069 | 0.698 | ++ |
| 0 | rs78038964 | 19 | 28479294 | intergenic | *LOC101927151,LOC100420587* | 0.153 | G | T | 0.829 | 1.62(1.20-2.18) | 1.8E-03 |  | 0.841 | 1.18(1.02-1.36) | 4.6E-03 |  | 0.839 | 1.25(1.10-1.42) | 6.8E-04 | 5.9E-02 | 0.061 | 0.714 | ++ |
| 0 | rs4805009 | 19 | 28480592 | intergenic | *LOC101927151,LOC100420587* | 0.151 | C | G | 0.826 | 1.61(1.20-2.17) | 1.8E-03 |  | 0.839 | 1.19(1.03-1.36) | 3.8E-03 |  | 0.837 | 1.25(1.10-1.42) | 4.7E-04 | 5.0E-02 | 0.067 | 0.703 | ++ |
| 0 | rs6120746 | 20 | 33448496 | intronic | *GGT7* | 0.434 | A | G | 0.558 | 1.37(1.10-1.71) | 4.8E-03 |  | 0.570 | 1.18(1.06-1.30) | 3.1E-03 |  | 0.568 | 1.21(1.10-1.33) | 7.5E-05 | 3.3E-03 | 0.210 | 0.363 | ++ |
| 0 | rs6119536 | 20 | 33450041 | intronic | *GGT7* | 0.434 | C | T | 0.558 | 1.37(1.10-1.71) | 4.8E-03 |  | 0.570 | 1.18(1.06-1.31) | 3.0E-03 |  | 0.568 | 1.21(1.10-1.33) | 7.2E-05 | 3.1E-03 | 0.212 | 0.357 | ++ |
| 1 | rs2064453 | 20 | 33460810 | upstream | *GGT7* | 0.437 | T | C | 0.559 | 1.39(1.11-1.73) | 3.6E-03 |  | 0.570 | 1.19(1.07-1.32) | 2.0E-03 |  | 0.568 | 1.22(1.11-1.34) | 3.0E-05 | 2.1E-03 | 0.209 | 0.365 | ++ |
| 1 | rs5999905 | 22 | 36038567 | intergenic | *MB,APOL6* | 0.057 | C | T | 0.954 | 2.44(1.35-4.43) | 3.6E-03 |  | 0.940 | 1.34(1.09-1.66) | 1.5E-03 |  | 0.943 | 1.43(1.18-1.75) | 3.8E-04 | 7.1E-02 | 0.063 | 0.710 | ++ |

| **Table S3. Analysis of 14 candidate risk variants in the third stage Validation population (GeneID-II) (3,274 cases/2,734 controls)** | | | | | | | | |
| --- | --- | --- | --- | --- | --- | --- | --- | --- |
| **Cytoband** | **SNP** | **CHR** | **Position** | **Genes** | **EA** | **EAF** | **OR (95%CI)** | ***P*** |
| 2q24.1 | rs6756964 | 2 | 158645538 | *ACVR1* | A | 0.787 | 1.06 (0.96-1.17) | 0.282 |
| 3q27.3 | rs3774268 | 3 | 186954324 | *MASP1* | G | 0.900 | 1.04 (0.90-1.20) | 0.638 |
| 4q27 | rs13103766 | 4 | 123517233 | *IL2,IL21* | G | 0.551 | 1.04 (0.95-1.14) | 0.367 |
| 4q32.2 | rs28587458 | 4 | 162424701 | *FSTL5* | T | 0.274 | 1.07 (0.97-1.17) | 0.197 |
| 5q33.2 | rs10041141 | 5 | 153097654 | *GRIA1* | T | 0.070 | 1.09 (0.92-1.29) | 0.325 |
| 5q33.2 | rs6580032 | 5 | 153110288 | *GRIA1* | A | 0.067 | 1.04 (0.87-1.24) | 0.640 |
| 6q24.1 | rs77334291 | 6 | 141196437 | *MIR4465,NMBR* | A | 0.846 | 1.02 (0.91-1.15) | 0.753 |
| 12q24.32 | rs10847208 | 12 | 127229506 | *LINC00943/LINC00944* | C | 0.842 | 1.20 (1.06-1.35) | 3.3x10^-3^ |
| 13q34 | rs74129462 | 13 | 112106861 | *TEX29,LINC00354* | G | 0.886 | 1.00 (0.88-1.14) | 0.994 |
| 14q32.11 | rs1287404 | 14 | 89983256 | *FOXN3* | G | 0.782 | 1.02 (0.92-1.13) | 0.724 |
| 15q24.1 | rs58232781 | 15 | 73105732 | *ADPGK-AS1,NEO1* | G | 0.541 | 1.01 (0.92-1.10) | 0.869 |
| 19q11 | rs2126982 | 19 | 28475962 | *LOC101927151* | G | 0.844 | 1.04 (0.93-1.18) | 0.466 |
| 20q11.22 | rs2064453 | 20 | 33460810 | *GGT7* | T | 0.558 | 1.14 (1.05-1.24) | 3.0x10^-3^ |
| 22q12.3 | rs5999905 | 22 | 36038567 | *MB,APOL6* | C | 0.941 | 1.02 (0.85-1.22) | 0.862 |
| Abbreviations: EA, effect allele; EAF, effect allele frequency; *P* is P value after adjustment with age, sex, age^2. | | | | | | | | |

| **Table S4. Genotypic association analysis between 14 SNPs and hypertension in the validation population (GeneID-II)** | | | | | |
| --- | --- | --- | --- | --- | --- |
| SNP | Genetic Model | Before adjustment | | After adjustment | |
|  |  | *P_obs_* | OR (95%CI) | *P_adj_* | OR (95%CI) |
| rs6756964 | Additive(AA/AG/GG) | 0.224 | 1.06(0.97-1.15) | 0.285 | 1.06(0.96-1.17) |
|  | Dominant(AA+AG/GG) | 0.441 | 1.10(0.86-1.40) | 0.645 | 1.07(0.81-1.41) |
|  | Recessive(AA/AG+GG) | 0.261 | 1.06(0.96-1.18) | 0.279 | 1.07(0.95-1.21) |
| rs3774268 | Additive(GG/AG/AA) | 0.668 | 1.03(0.91-1.16) | 0.646 | 1.03(0.90-1.19) |
|  | Dominant(GG+AG/AA) | 0.726 | 1.08(0.69-1.70) | 0.384 | 1.25(0.75-2.09) |
|  | Recessive(GG/AG+AA) | 0.560 | 1.04(0.91-1.19) | 0.805 | 1.02(0.87-1.19) |
| rs13103766 | Additive(GG/AG/AA) | 0.332 | 1.04(0.96-1.12) | 0.373 | 1.04(0.95-1.13) |
|  | Dominant(GG+AG/AA) | 0.606 | 1.04(0.91-1.18) | 0.773 | 1.02(0.88-1.19) |
|  | Recessive(GG/AG+AA) | 0.052 | 1.12(1.00-1.26) | 0.104 | 1.12(0.98-1.27) |
| rs28587458 | Additive(TT/AT/AA) | 0.744 | 1.01(0.93-1.10) | 0.211 | 1.06(0.97-1.17) |
|  | Dominant(TT+AT/AA) | 0.346 | 1.05(0.95-1.17) | 0.781 | 1.02(0.90-1.15) |
|  | Recessive(TT/AT+AA) | 0.016 | 1.26(1.04-1.52) | 8.13x10^-4^ | 1.45(1.17-1.80) |
| rs10041141 | Additive(TT/TC/CC) | 0.069 | 1.15(0.99-1.33) | 0.325 | 1.09(0.92-1.29) |
|  | Dominant(TT+TC/CC) | 0.159 | 1.12(0.96-1.31) | 0.562 | 1.06(0.88-1.26) |
|  | Recessive(TT/TC+CC) | 0.013 | 2.82(1.20-6.64) | 0.032 | 2.82(1.09-7.30) |
| rs6580032 | Additive(AA/AG/GG) | 0.504 | 1.05(0.90-1.23) | 0.639 | 1.04(0.87-1.25) |
|  | Dominant(AA+AG/GG) | 0.364 | 1.08(0.92-1.27) | 0.505 | 1.07(0.89-1.28) |
|  | Recessive(AA/AG+GG) | 0.303 | 1.55(0.67-3.58) | 0.369 | 1.57(0.59-4.20) |
| rs77334291 | Additive(AA/AG/GG) | 0.761 | 1.02(0.92-1.12) | 0.757 | 1.02(0.91-1.14) |
|  | Dominant(AA+AG/GG) | 0.209 | 1.23(0.89-1.70) | 0.307 | 1.21(0.84-1.74) |
|  | Recessive(AA/AG+GG) | 0.420 | 1.05(0.93-1.18) | 0.462 | 1.05(0.92-1.20) |
| rs10847208 | Additive(CC/CT/TT) | 0.011 | 1.14(1.03-1.26) | 3.43x10^-3^ | 1.19(1.06-1.34) |
|  | Dominant(CC+CT/TT) | 0.181 | 1.25(0.90-1.74) | 0.422 | 1.17(0.80-1.72) |
|  | Recessive(CC/CT+TT) | 6.95x10^-4^ | 1.22(1.09-1.38) | 3.01x10^-4^ | 1.28(1.12-1.47) |
| rs74129462 | Additive(GG/GT/TT) | 0.279 | 1.07(0.95-1.20) | 0.994 | 1.00(0.88-1.14) |
|  | Dominant(GG+GT/TT) | 0.666 | 1.10(0.71-1.70) | 0.098 | 1.50(0.93-2.42) |
|  | Recessive(GG/GT+TT) | 0.185 | 1.09(0.96-1.24) | 0.618 | 1.04(0.90-1.20) |
| rs1287404 | Additive(GG/AG/AA) | 0.548 | 1.03(0.94-1.13) | 0.724 | 1.02(0.92-1.13) |
|  | Dominant(GG+AG/AA) | 0.788 | 1.03(0.81-1.32) | 0.955 | 1.01(0.75-1.35) |
|  | Recessive(GG/AG+AA) | 0.397 | 1.05(0.94-1.17) | 0.690 | 1.03(0.90-1.16) |
| rs58232781 | Additive(GG/AG/AA) | 0.864 | 1.01(0.93-1.09) | 0.871 | 1.01(0.92-1.10) |
|  | Dominant(GG+AG/AA) | 0.562 | 1.04(0.91-1.19) | 0.896 | 1.01(0.87-1.17) |
|  | Recessive(GG/AG+AA) | 0.430 | 1.05(0.93-1.18) | 0.711 | 1.03(0.90-1.18) |
| rs2126982 | Additive(GG/GC/CC) | 0.133 | 1.08(0.98-1.20) | 0.467 | 1.05(0.93-1.18) |
|  | Dominant(GG+GC/CC) | 0.878 | 1.03(0.73-1.44) | 0.788 | 1.06(0.72-1.55) |
|  | Recessive(GG/GC+CC) | 0.078 | 1.11(0.99-1.25) | 0.357 | 1.07(0.93-1.22) |
| rs2064453 | Additive(TT/TC/CC) | 0.012 | 1.10(1.02-1.19) | 3.10x10^-3^ | 1.14(1.05-1.24) |
|  | Dominant(TT+TC/CC) | 0.649 | 1.03(0.90-1.18) | 0.340 | 1.08(0.92-1.26) |
|  | Recessive(TT/TC+CC) | 6.20x10^-4^ | 1.22(1.09-1.37) | 2.36x10^-4^ | 1.28(1.12-1.46) |
| rs5999905 | Additive(CC/CT/TT) | 0.946 | 1.01(0.86-1.17) | 0.865 | 1.02(0.85-1.22) |
|  | Dominant(CC+CT/TT) | 0.722 | 1.14(0.55-2.38) | 0.695 | 1.18(0.52-2.69) |
|  | Recessive(CC/CT+TT) | 0.878 | 1.01(0.86-1.20) | 0.784 | 1.03(0.85-1.25) |
| Additive model, (EA*EA / EA*NEA / NEA*NEA); Dominant model, (EA*EA + EA*NEA / NEA*NEA); Recessive model, (EA*EA / EA*NEA + NEA*NEA); EA, effect allele; Pobs is observed P value; Padj is P value after adjustment with age, sex, age^2.  Genotypic analysis assuming an additive, dominant or recessive model can reveal additional information about the specific genetic model for association of each variant with hypertension. SNP rs10847208 showed a more significant association under the recessive genetic model with *P*_rec-adj_ =3.01×10^-4^ (OR=1.28), and the same is true for rs2064453 with *P*_rec-adj_ =2.36×10^-4^ (OR=1.28) in the validation population (GeneID-II cohort). Note that SNP rs28587458 showed a significant genotypic association with hypertension under a recessive inheritance model (*P*_rec-adj_ = 8.13x10^-4^), although no allelic association was identified (Table S3). | | | | | |

| **Table S5. Significant association between two risk variants rs10847208 and rs2064453 and hypertension** | | | | | | | | |
| --- | --- | --- | --- | --- | --- | --- | --- | --- |
| **SNP** | **Population** | **N** | **EA** | **EAF** | ***OR*** | ***L95*** | ***U95*** | ***P*** |
| rs10847208 | GeneID-I | 685 | C | 0.84 | 1.58 | 1.18 | 2.13 | 2.6 x 10^-3^ |
|  | NHAPC | 2894 | C | 0.84 | 1.30 | 1.13 | 1.50 | 2.7 x 10^-4^ |
|  | GeneID-II | 6008 | C | 0.84 | 1.20 | 1.06 | 1.35 | 3.3 x 10^-3^ |
| rs2064453 | GeneID-I | 685 | T | 0.56 | 1.39 | 1.11 | 1.73 | 3.6 x 10^-3^ |
|  | NHAPC | 2894 | T | 0.57 | 1.19 | 1.07 | 1.32 | 2.0 x 10^-3^ |
|  | GeneID-II | 6008 | T | 0.56 | 1.14 | 1.05 | 1.24 | 3.0 x 10^-3^ |
| Abbreviations: EA, effect allele; EAF, effect allele frequency; *P* is P value after adjustment with age, sex, age^2. | | | | | | | | |

| **Table S6. Sequences of PCR primers and Taqman assay IDs** | |
| --- | --- |
| Primer Name | Sequence (5'-3') |
| rs10847208-HRM-F | TGGGGAAAACAGGTATTGGAAGT |
| rs10847208-HRM-R | CCACCAATGCAATCCACAAT |
| rs2064453-HRM-F | GCGCCTCTCCCAGCCCTCGG |
| rs2064453-HRM-R | CCCCGCGCTCTCCCCTCGAC |
| rs6580032-HRM-F | CATTGAGCAGCCTTGAAAGTAAA |
| rs6580032-HRM-R | GGTTTCATCAGTTCCTTCCTTCT |
| rs1287404-HRM-F | ACAGACAGGTCAAGGAGAGCAGTTC |
| rs1287404-HRM-R | ACAGGTGTGAGCCATTGGATCT |
| rs58232781-HRM-F | CTCTGGCAGGCTTCTTTTCATGT |
| rs58232781-HRM-R | CCAATCAGGTCCAAGCAAAGGTAA |
| rs6756964-taqman assay ID | C__29108693_20 |
| rs3774268-taqman assay ID | C__27499239_20 |
| rs13103766-taqman assay ID | ANMFXDJ |
| rs28587458-taqman assay ID | ANNKRXG |
| rs10041141-taqman assay ID | C_11275904_10 |
| rs77334291-taqman assay ID | ANZTFKD |
| rs74129462-taqman assay ID | ANTZ9M9 |
| rs2126982-taqman assay ID | C__15826343_10 |
| rs5999905-taqman assay ID | ANPRMJM |
| ACSS2-RT-F | CCACGAACGCTTTGAGACAACC |
| ACSS2-RT-R | ATCAATCCTGCCAGTGATCCAG |
| GSS-RT-F | CTTCAACCTGCTAGTGGATGCT |
| GSS-RT-R | ATGCCCTCTTTTAGGACTTGCTT |
| NCOA6-RT-F | GAAACTCCCGAACTGAAGAGC |
| NCOA6-RT-R | ATTTTCTTCGCTTGGATTGCAC |
| MAP1LC3A-RT-F | CCCCAGCAAAATCCCGGTGA |
| MAP1LC3A-RT-R | ATGATCTTGACCAACTCGCTCA |
| GGT7-RT-F | ACTTCAGCAATTACAGCGCCCTT |
| GGT7-RT-R | TCTGCCACCCAGTGAAGAGCCT |
| LINC00944-RT-F | AGCTGCTTCGGTCCCTGTAA |
| LINC00944-RT-R | CATTCCATTCCACAGTCTCTCTCTC |
| actin-RT-F | CGCGAGAAGATGACCCAGAT |
| actin-RT-R | TCACCGGAGTCCATCACGAT |
| ACVR2A-RT-F | GGGAGCTGCTGCAAAGTTGG |
| ACVR2A-RT-R | GGTTCTGTCTTTTTCCCAATTAGCA |
| CHMP1A-RT-F | GCCATGGACGATACCCTGTTC |
| CHMP1A-RT-R | GGCATACACACGGGCACACT |
| CREB1-RT-F | GCCACAGATTGCCACATTAG |
| CREB1-RT-R | TTGGACTTGTGGAGACTGAATAA |
| DLGAP4-RT-F | CCCCAAAAGGAAACTGTCAT |
| DLGAP4-RT-R | CCTGAACCCCGATGGACT |
| DYNLRB1-RT-F | TCGGAAATGGCAGAGGTGGAGGA |
| DYNLRB1-RT-R | GGGGGCCTGCTCCTGTCTCCAC |
| ERGIC3-RT-F | TTCAAGCAACGACTAGATAAAGATG |
| ERGIC3-RT-R | CAGGGAGTCAGGGTCAAACA |
| INO80-RT-F | TGGTGGTGCAGGATGTAAAGTAT |
| INO80-RT-R | CAAAAGCCGATTCCGACACT |
| MAPKAP1-RT-F | AAACCAGATCAAATGCAAAAATA |
| MAPKAP1-RT-R | AAGGGTTATTCAGCTGCAGAG |
| MTF1-RT-F | CCGGAAACAAAACGTAAAGA |
| MTF1-RT-R | GAGGCTGTAAGAGGTAAGGAA |
| PIGU-RT-F | GGCCTTTCACTGTTGGACTT |
| PIGU-RT-R | GCAAAATACAGGGCAATAGCA |
| PNKD-RT-F | GCGGGGGTGGGATCTGAACATG |
| PNKD-RT-R | GGGCCCGGGTCCTGTTATGAGAA |
| PPP6C-RT-F | TTGGAGACCTTCACTTACCTTC |
| PPP6C-RT-R | CTCATCTATTAAAGCTGCTACTGT |
| PRPF40B-RT-F | TCTATGTGGAGGAGTTGAAGGCACG |
| PRPF40B-RT-R | TCCAGTGCGGCAGCCCTCT |
| UQCC1-RT-F | AAGCCATGGGATTCACGGGACCT |
| UQCC1-RT-R | GCCGCGCTGCTGAACATCCTC |
